# Event-Based Update of Synapses in Voltage-Based Learning Rules

**DOI:** 10.3389/fninf.2021.609147

**Published:** 2021-06-10

**Authors:** Jonas Stapmanns, Jan Hahne, Moritz Helias, Matthias Bolten, Markus Diesmann, David Dahmen

**Affiliations:** ^1^Institute of Neuroscience and Medicine (INM-6), Institute for Advanced Simulation (IAS-6), JARA Institute Brain Structure Function Relationship (INM-10), Jülich Research Centre, Jülich, Germany; ^2^Department of Physics, Institute for Theoretical Solid State Physics, RWTH Aachen University, Aachen, Germany; ^3^School of Mathematics and Natural Sciences, Bergische Universität Wuppertal, Wuppertal, Germany; ^4^Department of Physics, Faculty 1, RWTH Aachen University, Aachen, Germany; ^5^Department of Psychiatry, Psychotherapy and Psychosomatics, Medical Faculty, RWTH Aachen University, Aachen, Germany

**Keywords:** event-based simulation, voltage-based plasticity rules, spiking neural network simulator, NEST, Clopath rule, Urbanczik-Senn rule

## Abstract

Due to the point-like nature of neuronal spiking, efficient neural network simulators often employ event-based simulation schemes for synapses. Yet many types of synaptic plasticity rely on the membrane potential of the postsynaptic cell as a third factor in addition to pre- and postsynaptic spike times. In some learning rules membrane potentials not only influence synaptic weight changes at the time points of spike events but in a continuous manner. In these cases, synapses therefore require information on the full time course of membrane potentials to update their strength which a priori suggests a continuous update in a time-driven manner. The latter hinders scaling of simulations to realistic cortical network sizes and relevant time scales for learning. Here, we derive two efficient algorithms for archiving postsynaptic membrane potentials, both compatible with modern simulation engines based on event-based synapse updates. We theoretically contrast the two algorithms with a time-driven synapse update scheme to analyze advantages in terms of memory and computations. We further present a reference implementation in the spiking neural network simulator NEST for two prototypical voltage-based plasticity rules: the Clopath rule and the Urbanczik-Senn rule. For both rules, the two event-based algorithms significantly outperform the time-driven scheme. Depending on the amount of data to be stored for plasticity, which heavily differs between the rules, a strong performance increase can be achieved by compressing or sampling of information on membrane potentials. Our results on computational efficiency related to archiving of information provide guidelines for the design of learning rules in order to make them practically usable in large-scale networks.

## 1. Introduction

One mechanism for learning in the brain is implemented by changing the strengths of connections between neurons, known as synaptic plasticity. Already early on, such plasticity was found to depend on the activity of the connected neurons. Donald Hebb postulated the principle “Cells that fire together, wire together” (Hebb, 1949). Later on, it was shown that plasticity is shaped by temporal coordination of activities even down to the level of individual spikes (Markram et al., 1997; Bi and Poo, 1998). Synaptic plasticity rules for spiking neural networks, such as spike timing-dependent plasticity (STDP, Gerstner et al., 1996), consequently employ spike times of pre- and postsynaptic cells to predict the change in connections.

In recent years, a new class of biologically inspired plasticity rules has been developed that takes into account the membrane potential of the postsynaptic neuron as an additional factor (for a review, see Mayr and Partzsch, 2010; Gerstner et al., 2014). The rule by Clopath et al. (2010) can be seen as a prototypical example for a voltage-based plasticity rule since long-term potentiation of synapses depends on the presynaptic spike arrival and a filtered version of the postsynaptic membrane potential. This additional voltage dependence enables the Clopath rule to describe phenomena that are not covered by ordinary STDP but can be observed in experimental data, such as the complex frequency dependence of the synaptic weight changes in spike pairing experiments (Sjöström et al., 2001). Furthermore, it provides a mechanism for the creation of strong bidirectional connections in networks, which have been found to be overrepresented in some cortical areas (Song et al., 2005).

Further inspiration for recently proposed plasticity rules originates from the field of artificial neural networks. These networks showed great success in the past decade, for example in image or speech recognition tasks (Hinton et al., 2006; Krizhevsky et al., 2012; Hannun et al., 2014; LeCun et al., 2015). The involved learning paradigms, for example the backpropagation algorithm (Werbos, 1974; Lecun, 1985; Parker, 1985; Rumelhart et al., 1986), are, however, often not compatible with biological constraints such as locality of information for weight updates. To bridge the gap to biology, different biologically inspired approximations and alternatives to the backpropagation algorithm have been proposed (Neftci et al., 2017; Sacramento et al., 2018; Bellec et al., 2020; Cartiglia et al., 2020). A common feature of many of these rules is that weight updates not only depend on the output activity of pre- and postsynaptic cells, but also on a third factor, which is a time-continuous signal. A prominent example of such biologically and functionally inspired rules is the voltage-based plasticity rule proposed by Urbanczik and Senn (2014), where the difference between somatic and dendritic membrane potential serves as an error signal that drives learning. This rule, incorporated in complex microcircuits of multi-compartment neurons, implements local error-backpropagation (Sacramento et al., 2018).

Research on functionally inspired learning rules in biological neural networks is often led by the requirement to implement a particular function rather than efficiency. Present studies are therefore primarily designed to prove that networks with a proposed learning rule minimize a given objective function. Indeed many learning rules are rather simple to implement and to test in *ad-hoc* implementations where at any point the algorithm has access to all state variables. While the latter implementations are sufficient for a proof of principle, they are hard to reuse, reproduce, and generalize. In particular, simulations are restricted to small network sizes, as the simulation code cannot be straight-forwardly distributed across compute nodes and thus parallelized. This also limits the simulation speed which is, in particular, problematic given that successful learning requires simulating networks for long biological times.

In parallel to the above efforts are long-term developments of simulation software for biological neural networks (for a review, see Brette et al., 2007). Such open-source software, combined with interfaces and simulator-independent languages (Davison et al., 2008; Djurfeldt et al., 2010, 2014), supports maintainability and reproducibility, as well as community driven development. The design of such simulators is primarily led by implementation efficiency. Code is optimized for neuron and synapse dynamics, with the aim to upscale simulations to biologically realistic network sizes. A modular structure of the code facilitates re-use and extensions in functionality. Therefore, one aim of the community should be the transfer of *ad-hoc* proof-of-principle implementations to these well-tested platforms. Given the differences in design principles behind the exploratory development of specific models and general-purpose simulation technology, this transfer is not trivial. In the current study, we show how to make voltage-based learning rules compatible with spiking neural network simulators that employ an event-driven update scheme for synapses.

**Figure 1:**
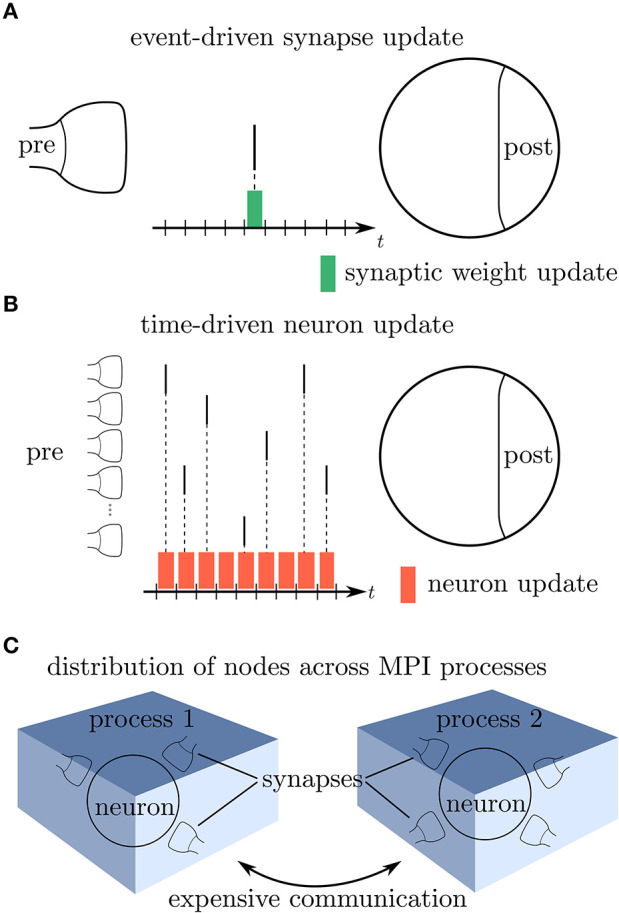
Update schemes for neurons and synapses. **(A)** A spike crosses a synapse from the presynaptic (pre) to the postsynaptic (post) neuron. Since this is a rare event, the synaptic weight is computed only when a spike is delivered, indicated by the green bar (event-driven update). **(B)** Neurons with a large in-degree receive spikes in rapid succession which suggests a time-driven update of the neuron's state in each time step (red bars). **(C)** Since the computation of the synaptic weights requires information from the postsynaptic neuron, storing the synapses on the same compute node reduces the amount of expensive communication between compute processes.

Modern network simulators use individual objects for different neurons and synapses. One common strategy of parallelization is to distribute these objects across many compute processes (Lytton et al., 2016; Jordan et al., 2018). Communication between neurons then implies exchange of information between compute processes. Neurons in the brain primarily communicate in an event-based fashion via spikes. The duration of these spike events is on the order milliseconds, which together with typical rates during physiological brain states of a few spikes per second yields a coupling that is sparse in time (Figure 1A). Spiking simulators emulate this communication by idealizing spikes as instantaneous events. Thus, in the absence of direct electrical coupling via gap junctions (Kumar and Gilula, 1996; Hahne et al., 2015; Jordan et al., 2020a), there is no neuronal interaction in between two spike events such that the dynamics of neuronal and synaptic state variables can be evolved independently in time. This led to the development of event-based simulation schemes, where synapses are only updated in their state at the times of incoming spikes (Watts, 1994; Morrison et al., 2005). This significantly reduces the amount of function calls to synapse code and optimizes computational performance in network simulations. Modern spiking network simulators such as Auryn (Zenke and Gerstner, 2014), Brian2 (Stimberg et al., 2014), Neuron (Carnevale and Hines, 2006), NEST (Gewaltig and Diesmann, 2007), and Nevesim (Pecevski et al., 2014) therefore employ an event-based update scheme for synapses. Even though spike events at single synapses are rare, each single neuron typically receives a large amount of spikes in rapid succession due to its large number of incoming connections (in-degree). This suggests a time-driven update of neurons (Figure 1B). The resulting hybrid simulation scheme for neurons and synapses (Morrison et al., 2005; D'Haene et al., 2014; Krishnan et al., 2017) is nowadays commonly used across many spiking network simulators (for a review, see Brette et al., 2007).

An event-based scheme for synapses is perfectly suitable for classical STDP rules, which only rely on a comparison between the timings of spike events. In these rules, synaptic weights formally depend on spike traces, which are continuous signals that are fully determined by spike timings of pre- and postsynaptic neurons and which can be updated at the time of spike events. Optimizations of simulations including STDP have been extensively discussed (Song et al., 2000; Ros et al., 2006; Rudolph and Destexhe, 2006; Morrison et al., 2007a) and routinely used in spiking network simulators such as Auryn (Zenke and Gerstner, 2014), Brian2 (Stimberg et al., 2014), Neuron (Carnevale and Hines, 2006), NEST (Gewaltig and Diesmann, 2007), and Nevesim (Pecevski et al., 2014) as well as in neuromorphic hardware (Pfeil et al., 2013; Serrano-Gotarredona et al., 2013; Neftci et al., 2014; Galluppi et al., 2015; Friedmann et al., 2016; Thakur et al., 2018). Some STDP variants also include the membrane potential of postsynaptic cells at the time points of presynaptic spike events as a gating variable (Brader et al., 2007; Diederich et al., 2018). At the update, these rules only require the synapse to know the current value of the postsynaptic membrane potential in addition to the pre- and postsynaptic spike time. Obtaining this value from the neuron objects is efficient to implement and already employed in Brian2 (Stimberg et al., 2014) and in a range of neuromorphic systems (Serrano-Gotarredona et al., 2013; Galluppi et al., 2015; Qiao et al., 2015; Moradi et al., 2018; Cartiglia et al., 2020).

We here focus on more complex voltage-based learning rules which not only rely on membrane potentials at the time points of spike events, but on an extended history of membrane potentials. For these rules synapses continuously require information from the postsynaptic neurons in order to update their weights (Clopath et al., 2010; Mayr and Partzsch, 2010; Brea et al., 2013; Yger and Harris, 2013; Urbanczik and Senn, 2014; Albers et al., 2016). This a priori breaks the idea behind an event-based update scheme. Therefore, previous attempts to incorporate such voltage-based plasticity in spiking network simulators resorted to time-driven synapse updates for NEST (Jordan et al., 2020b) and NEURON (see implementation of Clopath plasticity on ModelDB, Hines et al., 2004). These implementations therefore only profit from the simulation environment on the level of the implementation language, but have not been able to exploit the algorithmic optimizations and speedup of event-based synapse updates.

In this study we present an efficient archiving method for the history of postsynaptic state variables that allows for an event-based update of synapses and thus makes complex voltage-based plasticity rules compatible with state-of-the-art simulation technology for spiking neural networks. In particular, we derive two event-based algorithms that store a time-continuous or discontinuous history, respectively. These algorithms apply to plasticity rules with any dependence on post-synaptic state variables and therefore cover a large range of existing models (Brader et al., 2007; Mayr and Partzsch, 2010; Legenstein and Maass, 2011; Brea et al., 2013, 2016; Yger and Harris, 2013; Qiao et al., 2015; Albers et al., 2016; Sheik et al., 2016; Diederich et al., 2018; Sacramento et al., 2018; Cartiglia et al., 2020). We theoretically analyze advantages of the two event-driven algorithms with respect to each other and compare to a straight-forward time-driven algorithm.

The presented simulation concepts are exemplified and evaluated in a reference implementation in the open source simulation code NEST (Gewaltig and Diesmann, 2007; Jordan et al., 2019). The reference implementation thereby exploits existing functionality of a scalable software platform which can be used on laptops as well as supercomputers. NEST is employed by a considerable user community and equipped with an interface to the programming language Python (Eppler et al., 2009) that is currently widely used in the field of computational neuroscience (Muller et al., 2015). It supports relevant neuron models and connection routines for the construction of complex networks. Despite this flexibility the simulation engine shields the researcher from the difficulties of handling a model description in a distributed setting (Morrison et al., 2005; Plesser et al., 2015).

To exemplify the general simulation algorithms, we here focus on the voltage-based plasticity rules by Clopath et al. (2010) and Urbanczik and Senn (2014). The two rules represent opposing ends of a family of learning rules in the amount of data required to compute weight updates. The Clopath rule by design only triggers plasticity in the vicinity of postsynaptic spike events; storing a history, which is non-continuous in time, thus becomes beneficial. In contrast, the Urbanczik-Senn rule considers noisy prediction errors based on postsynaptic membrane voltages and spikes. Such prediction errors never vanish and therefore always need to be stored to update the weights, leading to time-continuous histories. For a given span of biological time, simulations of the Urbanczik-Senn rule are therefore by design less efficient than those of the Clopath rule. However, we show that a compression of membrane potential information reduces this performance gap. Changing the learning rule to include a sparse sampling of the membrane voltage further increases efficiency and makes performance comparable to simulations with ordinary STDP.

Our study begins with a specification of the mathematical form of the learning rules that we consider (section 2.1). We distinguish between classical STDP (section 2.2) and voltage-based rules (section 2.3) and present a special case where voltage-based rules can be efficiently implemented by compressing information on the postsynaptic membrane potential. We then introduce the Clopath and the Urbanczik-Senn rule as two examples of voltage-based plasticity (sections 2.4 and 2.5). In section 3 we first contrast time- and event-driven schemes for updating synapses with voltage-based plasticity (section 3.1). Subsequently, we detail a reference implementation of the algorithms in NEST (section 3.2) and use this to reproduce results from the literature (section 3.3). After that, we examine the performance of the reference implementation for the Clopath and the Urbanczik-Senn rule (section 3.4). Conclusions from the implementation of the two rules are drawn in section 3.5, followed by a general Discussion in section 4. The technology described in the present article is available in the 2.20.1 release of the simulation software NEST as open source. The conceptual and algorithmic work is a module in our long-term collaborative project to provide the technology for neural systems simulations (Gewaltig and Diesmann, 2007).

## 2. Materials and Methods

### 2.1. General Structure of Learning Rules

**Figure 2:**
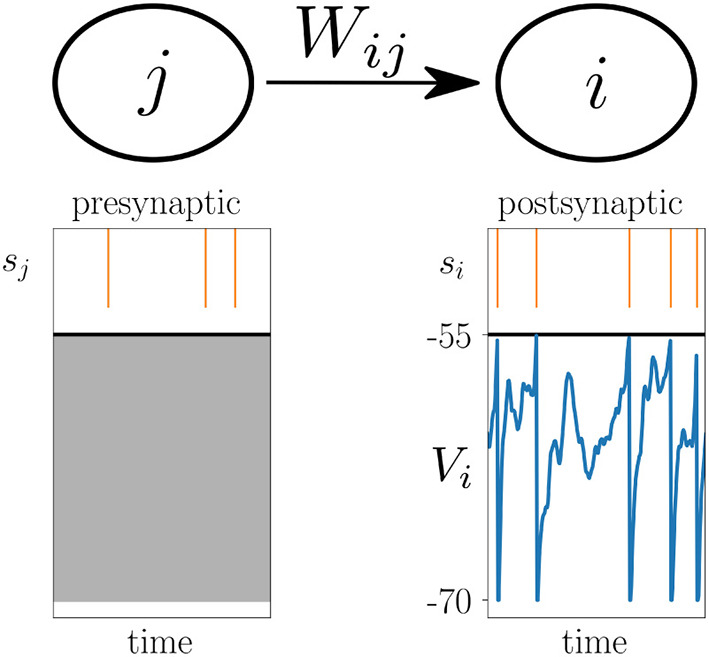
Voltage-based plasticity rules. The change Δ*W*_*ij*_ in synaptic strength between presynaptic neuron *j* and postsynaptic neuron *i* depends on the presynaptic spike train *s*_*j*_, the postsynaptic spike train *s*_*i*_ and the postsynaptic membrane potential *V*_*i*_.

The focus of this study are plasticity models of the general form

(1) $$\begin{array}{l} \frac{dW_{ij}\left(t\right)}{dt}=F\left(W_{ij}\left(t\right),s_{i}^{*}\left(t\right),s_{j}^{*}\left(t\right),V_{i}^{*}\left(t\right)\right), \end{array}$$

where the change $\frac{dW_{ij}\left(t\right)}{dt}$ of the synaptic weight *W*_*ij*_ between the presynaptic neuron *j* and postsynaptic neuron *i* is given by a function *F* that potentially depends on the current synaptic weight *W*_*ij*_(*t*), as well as on $s_{i}^{*}\left(t\right),s_{j}^{*}\left(t\right),V_{i}^{*}\left(t\right)$ which are causal functionals of the postsynaptic spike train *s*_*i*_, the presynaptic spike train *s*_*j*_, and the postsynaptic membrane potential *V*_*i*_, respectively (Figure 2). Causal functional here refers to $s_{i}^{*}\left(t\right)$ potentially depending on all past values $s_{i}\left(t^{\prime }\leq t\right)$; likewise V^*^(*t*) depends on *V*(*t*′ ≤ *t*). Note that for simplicity of the notation, we only show one function *F* on the right hand side of (1), while generally there could be a sum of multiple functions or functionals *F*_α_, where each one depends on spike trains and membrane potentials in a different manner. Note also that *F* mixes information of pre- and postsynaptic neurons, while the functionals denoted by * only need to take into account information of either the pre- or postsynaptic neuron. In cases where *F* is a functional, i.e., where *F* depends on the whole time course of its arguments, it can take into account an additional joint history dependence on $s_{i}^{*},s_{j}^{*}$, and $V_{i}^{*}$. A special case, the Urbanczik-Senn learning rule, is discussed further below.

One can formally integrate (1) to obtain the weight change between two arbitrary time points *t* and *T*

(2) $$\begin{array}{l} \Delta W_{ij}\left(t,T\right)=\int_{t}^{T}dt^{\prime }F\left(W_{ij}\left(t^{\prime }\right),s_{i}^{*}\left(t^{\prime }\right),s_{j}^{*}\left(t^{\prime }\right),V_{i}^{*}\left(t^{\prime }\right)\right). \end{array}$$

### 2.2. Spike-Timing Dependent Plasticity

In general, the integral on the right hand side of the equation cannot be calculated analytically. There is, however, a notable exception, which is the model of spike-timing dependent plasticity (STDP). This model is a form of Hebbian plasticity that relies on the exact spike times of pre- and postsynaptic neurons and ignores any effect of the postsynaptic membrane potential. The dependence on the exact spike times becomes apparent by the fact that either the pre- or postsynaptic spike functional is the spike train itself, for example

(3) $$\begin{array}{l} s_{i}^{*}\left(t\right)=s_{i}\left(t\right)=\underset{k}{\sum }\delta \left(t-t_{i}^{k}\right), \end{array}$$

where $t_{i}^{k}$ is the *k*-th spike of the *i*-th neuron. This yields a plasticity rule that reads (Morrison et al., 2008)

(4) $$\begin{array}{l} \frac{dW_{ij}\left(t\right)}{dt}=-f_{-}\left(W_{ij}\left(t\right)\right)s_{-,i}^{*}\left(t\right)s_{j}\left(t\right)+f_{+}\left(W_{ij}\left(t\right)\right)s_{+,j}^{*}\left(t\right)s_{i}\left(t\right) \end{array}$$

with functions *f*_±_ that model the weight dependence, and functionals $s_{\pm }^{*}\left(t\right)=\left(\kappa_{\pm }*s\right)\left(t\right)$ given as convolutions of spike trains with kernels κ_±_, which in the classical STDP rule correspond to one-sided exponential decays. The appearance of the raw spike trains (delta distributions) in the differential equation of the STDP model renders the integration of the ODE trivial

(5) $$\begin{array}{l} \Delta W_{ij}\left(t,T\right)=-\underset{\text{spikes }k}{\sum }f_{-}\left(W_{ij}\left(t_{j}^{k}\right)\right)\kappa_{-,i}\left(t_{j}^{k}\right)\\ \;+\underset{\text{spikes }l}{\sum }f_{+}\left(W_{ij}\left(t_{i}^{l}\right)\right)\kappa_{+,j}\left(t_{i}^{l}\right), \end{array}$$

where $t_{j}^{k},t_{i}^{l}\in \left[t,T\right]$. An update of the synaptic weight between any two time points only requires knowledge of the weight and spike functionals at the timing of the pre- and postsynaptic spikes.

For models that do not solely rely on exact spike times, but for example on filtered versions of the spike trains, much more information is needed in order to calculate a weight update Δ*W*_*ij*_(*t, T*) between any two time points. This makes the computation more involved: the synapse needs all values of $W_{ij}\left(t^{\prime }\right),s_{i}^{*}\left(t^{\prime }\right),s_{j}^{*}\left(t^{\prime }\right),V_{i}^{*}\left(t^{\prime }\right)$ for *t*′ ∈ [*t, T*] to update its weight. The remainder of this study describes different approaches to this problem and their advantages and disadvantages.

### 2.3. Voltage-Based Plasticity

In a time-driven neuron update, the membrane potential in many simulators is computed at each simulation step *t*^α^ = α · *h*, where *h* is the simulation step size and α ∈ ℕ. For plasticity models that rely on the membrane potential, the time discretization of (2) therefore yields

(6) $$\begin{array}{l} \Delta W_{ij}\left(t,T\right)=\underset{\text{steps }\alpha }{\sum }\Delta W_{ij}\left(t^{\alpha },t^{\alpha +1}\right), \end{array}$$

(7) $$\begin{array}{l} \Delta W_{ij}\left(t^{\alpha },t^{\alpha +1}\right)=\int_{t^{\alpha }}^{t^{\alpha +1}}dt^{\prime }F\left(W_{ij}\left(t^{\prime }\right),s_{i}^{*}\left(t^{\prime }\right),s_{j}^{*}\left(t^{\prime }\right),V_{i}^{*}\left(t^{\alpha }\right)\right). \end{array}$$

which, in comparison to the small sum over spikes in the STDP rule (5), contains a large sum over all time steps *t*^α^ in between time points *t* and *T*. As the membrane potential is only known at time points *t*′ = *t*^α^, it generally enters (7) in a piecewise constant manner – hence the argument *V*(*t*^α^). The synapse therefore predominantly needs information of the postsynaptic neuron in order to update its weight. Thus, in a distributed simulation framework, where neurons are split across multiple compute processes, it is beneficial to store the synapses at the site of the postsynaptic neurons in order to reduce communication (Figure 1C). This confirms the earlier design decision of Morrison et al. (2005) who place synapses at the site of the postsynaptic neuron to reduce the amount of data communicated by the presynaptic site.

If weight changes Δ*W*_*ij*_ depend on the synaptic weight themselves, then (7) cannot be used in practice as intermediate weights $W_{ij}\left(t^{\prime }\right)$ for *t*^α^ < *t*′ < *t*^α+1^ are not known. In this scenario, weight changes have to be calculated on the simulation grid with $W_{ij}\left(t^{\prime }\right)\to W_{ij}\left(t^{\alpha }\right)$ in case of a forward Euler scheme, or $W_{ij}\left(t^{\prime }\right)\to W_{ij}\left(t^{\alpha +1}\right)$ in case of a backward Euler scheme. In the following we, for simplicity, stick to the forward Euler setting and arrive at the core computation for voltage-based plasticity rules

(8) $$\begin{array}{l} \Delta W_{ij}\left(t^{\alpha },t^{\alpha +1}\right)=\int_{t^{\alpha }}^{t^{\alpha +1}}dt^{\prime }F\left(W_{ij}\left(t^{\alpha }\right),s_{i}^{*}\left(t^{\prime }\right),s_{j}^{*}\left(t^{\prime }\right),V_{i}^{*}\left(t^{\alpha }\right)\right). \end{array}$$

Given that *s*_*i*_ and *s*_*j*_ are spike trains, the functionals $s_{i}^{*}$ and $s_{j}^{*}$ are obtained trivially from the kernels of their corresponding Volterra expansions. If *F* in addition does not depend on $s_{i}^{*}$ and $s_{j}^{*}$ in a too complicated manner, which is usually the case (see examples below), the integral in (8) can be calculated analytically.

#### 2.3.1. Compression of Postsynaptic Information

The major operation of the plasticity scheme in terms of frequency and complexity is the computation of infinitesimal weight changes $\Delta W_{ij}\left(t^{\alpha },t^{\alpha +1}\right)$. Since the presynaptic spike train $s_{j}^{*}$ enters *F* in (8), the same postsynaptic information on $s_{i}^{*}$ and $V_{i}^{*}$ is used many times for very similar computations: the membrane potential trace of each neuron is effectively integrated many times. Is there a way to employ the result of the computation $\Delta W_{ij}\left(t^{\alpha },t^{\alpha +1}\right)$ for neuron *j* for the computations $\Delta W_{ik}\left(t^{\alpha },t^{\alpha +1}\right)$ for other neurons *k* ≠ *j*? In a simple setting, where *F* factorizes into $F\left(W_{ij}\left(t\right),s_{i}^{*}\left(t\right),s_{j}^{*}\left(t\right),V_{i}^{*}\left(t\right)\right)=s_{j}^{*}\left(t\right)G\left(s_{i}^{*}\left(t\right),V_{i}^{*}\left(t\right)\right)$ with $s_{j}^{*}\left(t\right)=\left(\kappa *s_{j}\right)\left(t\right)$ and

(9) $$\begin{array}{l} \kappa \left(t\right)=H\;\left(t\right)\frac{1}{\tau }e^{-\frac{t}{\tau }}, \end{array}$$

defined via the Heaviside step function *H*(*x*), we can make use of the property

(10) $$\begin{array}{l} s_{j}^{*}\left(t\right)=\left(s_{j}^{*}\left(t_{LS}\right)+\tau^{-1}\right)\;e^{-\left(t-t_{LS}\right)/\tau }, \end{array}$$

where *t* > *t*_*LS*_ and *t*_*LS*_ denotes the last spike time of the presynaptic neuron. In this case the weight update in between two spike events factorizes

(11) $$\begin{array}{l} \Delta W_{ij}\left(t_{LS},t_{\text{S}}\right)=\underset{=:\;{\overset{-}{x}}_{j}\left(t_{LS}\right)}{\underset{︸}{\left(s_{j}^{*}\left(t_{LS}\right)+\tau^{-1}\right)}}\underset{=:\;\Delta W_{i}\left(t_{LS},t_{\text{S}}\right)}{\underset{︸}{\int_{t_{LS}}^{t_{\text{S}}}dt^{\prime }e^{-\left(t^{\prime }-t_{LS}\right)/\tau }G\left(s_{i}^{*}\left(t^{\prime }\right),V_{i}^{*}\left(t^{\prime }\right)\right)}}, \end{array}$$

where the latter integral Δ*W*_*i*_(*t*_*LS*_, *t*_S_) is independent of the presynaptic spike train $s_{j}^{*}$. Moreover, Δ*W*_*i*_ depends on *t*_*LS*_ only via an exponential prefactor. Thus, an integral Δ*W*_*i*_(*t*_1_, *t*_2_) over an arbitrary time interval *t*_LS_ ≤ *t*_1_ < *t*_2_ ≤ *t*_*S*_ which is completely independent of any presynaptic information, can be used as a part of the whole integral Δ*W*_*i*_(*t*_*LS*_, *t*_S_) since it can be decomposed as

$$\begin{array}{l} \Delta W_{i}\left(t_{LS},t_{\text{S}}\right)=\Delta W_{i}\left(t_{LS},t_{1}\right)+e^{-\frac{t_{1}-t_{\text{LS}}}{\tau }}\Delta W_{i}\left(t_{1},t_{\text{2}}\right)\\ \;+e^{-\frac{t_{2}-t_{\text{LS}}}{\tau }}\Delta W_{i}\left(t_{2},t_{S}\right). \end{array}$$

Therefore, whenever an integral of the postsynaptic quantities $s_{i}^{*}$ and $V_{i}^{*}$ is computed, it can be used to advance the weight update of all incoming connections and the integration only needs to be performed once. To account for the generally different last spike times *t*_LS_ of the incoming connections, the postsynaptic neuron stores the different Δ*W*_*i*_(*t*_*LS*_, *t*) in a so-called *compressed history*. At the time of an incoming spike event, Δ*W*_*i*_(*t*_*LS*_, *t*_S_) can be read out by the synapse for the correct *t*_*LS*_ of that synapse and be combined with the stored presynaptic spike trace $s_{j}^{*}$.

### 2.4. Example 1: Clopath Plasticity

The Clopath rule (Clopath et al., 2010) was designed as a voltage-based STDP rule that accounts for non-linear effects of spike frequency on weight changes which had been previously observed in experiments (Sjöström et al., 2001). It does so by using the evolution of the postsynaptic membrane voltage around postsynaptic spike events instead of the postsynaptic spikes themselves. This requires a neuron model that takes into account features of membrane potential excursions near spike events, such as modified adaptive exponential integrate-and-fire (aeif) model neurons that are used in the original publication (Clopath et al., 2010, see section 5.2) or Hodgkin-Huxley (hh) neurons that are used in a NEURON reference implementation on ModelDB (Hines et al., 2004).

The plasticity rule is of the general form (1) with a sum of two different functions *F*_α_ on the right hand side. It treats long-term depression (LTD) and potentiation (LTP) of the synaptic weight in the two terms *F*_LTD_ and *F*_LTP_, with

(12) $$\begin{array}{l} F_{\text{LTD}}\;(s_{j}\left(t\right),V_{i,\text{LTD}}^{*}\left(t\right))=-A_{\text{LTD}}\;s_{j}\left(t\right)\;V_{i,\text{LTD}}^{*}\left(t\right)\\ \text{ with }V_{i,\text{LTD}}^{*}={\left({ū}_{-}-\theta_{-}\right)}_{+},\\ \;{ū}_{-}\left(t\right)=\left(\kappa_{-}*V_{i}\right)\left(t-d_{s}\right) \end{array}$$

and

(13) $$\begin{array}{l} F_{\text{LTP}}\left(s_{j}^{*}\left(t\right),V_{i,\text{LTP}}^{*}\left(t\right)\right)=A_{\text{LTP}}\;s_{j}^{*}\left(t\right)\;V_{i,\text{LTP}}^{*}\left(t\right)\\ \text{ with }s_{j}^{*}=\kappa_{s}*s_{j},\\ \;V_{i,\text{LTP}}^{*}={\left({ū}_{+}-\theta_{-}\right)}_{+}{\left(V_{i}-\theta_{+}\right)}_{+},\\ \;{ū}_{+}\left(t\right)=\left(\kappa_{+}*V_{i}\right)\left(t-d_{s}\right). \end{array}$$

**Figure 3:**
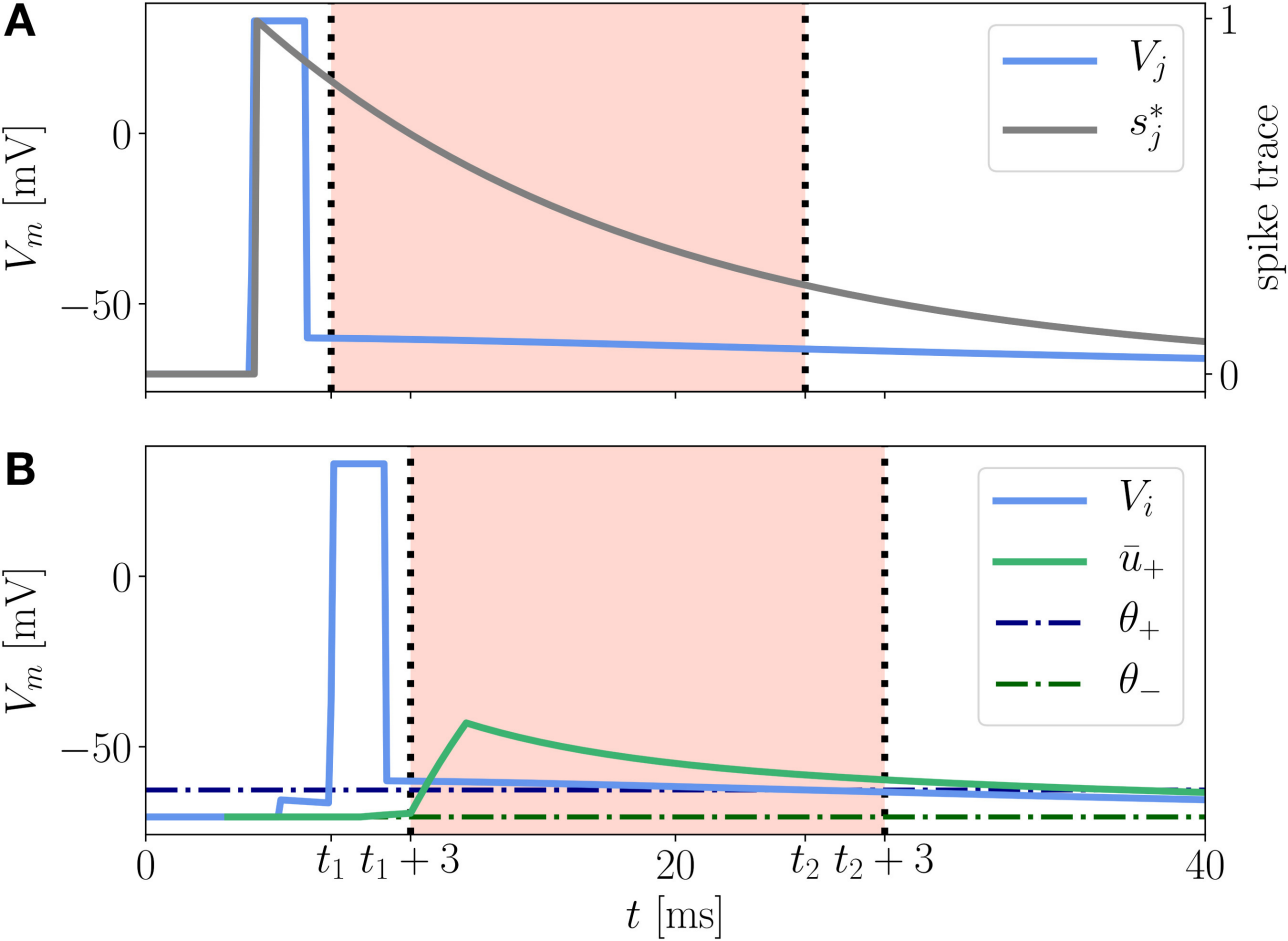
Illustration of LTP contribution to the Clopath rule. A presynaptic neuron **(A)** and a postsynaptic neuron **(B)** emit a spike at *t*_sp,pre_ = 4 ms and *t*_sp,post_ = 6 ms, respectively. The presynaptic spike elicits a trace $s_{j}^{*}$ (gray) at the synapse. The excursion of the postsynaptic membrane potential *V*_*i*_ (**B**, blue) elevates the low-pass filtered potential ū_+_ (green) so that both *V*_*i*_ and ū_+_ exceed the respective thresholds θ_+_ (dash-dotted, dark blue) and θ_−_ (dash-dotted, dark green), cf. (13), between *t*_1_ and *t*_2_. Only within this period, shifted by *d*_*s*_ = 3 ms, which is for times *t*_1_ + 3 ms < *t* < *t*_2_ + 3 ms (**B**, red background), see section 3.2.3 for details, the LTP of the synaptic weight is non-vanishing because of the threshold-linear functions in equation (13). The shift by *d*_*s*_ = 3 ms does not apply to the spike trace (**A**, red background). The rectangular shape of the spikes is achieved by a clamping of the membrane potential to *V*_clamp_ = 33 mV for a period of *t*_clamp_ = 2 ms.

Here (*x* − *x*_0_)_+_ = *H*(*x* − *x*_0_)(*x* − *x*_0_) is the threshold-linear function and *H*(*x*) is the Heaviside step function. *A*_LTD_ and *A*_LTP_ are prefactors controlling the relative strength of the two contributions. κ_±_ are exponential kernels of the form (9), which are applied to the postsynaptic membrane potential, and κ_*s*_ is an exponential kernel applied to the presynaptic spike train. The time-independent parameters θ_±_ serve as thresholds below which the (low-pass filtered) membrane potential does not cause any weight change (Figure 3). Note that *A*_LTP_ can also depend on the membrane potential. This case is described in Appendix Section 5.5.

In a reference implementation of the Clopath rule by C. Clopath and B. Torben-Nielsen available on ModelDB (Hines et al., 2004), there is a subtle detail not explicitly addressed in the original journal article. In their implementation the authors introduce an additional delay *d*_*s*_ between the convolved version of the membrane potentials ū_±_ and the bare one [cf. parameter *d*_*s*_ in (12) and (13)]. The convolved potentials are shifted backwards in time by the duration of a spike *d*_*s*_ (see Supplementary Tables 1, 3). As a result, the detailed shape of the excursion of the membrane potential during a spike of the postsynaptic neuron does not affect the LTP directly but only indirectly via the low-pass filtered version ū_+_, see red background in Figure 3B. Incorporating this time shift in ū_±_ is essential to reproduce the results from Clopath et al. (2010) on spike-pairing experiments.

The depression term *F*_LTD_ depends on the unfiltered spike train *s*_*j*_. It can thus be treated analogous to ordinary STDP rules (cf. (4)ff). In particular, $V_{i,\text{LTD}}^{*}$ only needs to be available for time points of presynaptic spikes (potentially taking into account additional delays of the connection). The potentiation term *F*_LTP_, however, depends on the filtered spike train $s_{j}^{*}$; $V_{i,\text{LTP}}^{*}$ consequently needs to be known also for times in between spike events.

### 2.5. Example 2: Urbanczik-Senn Plasticity

The Urbanczik-Senn rule (Urbanczik and Senn, 2014) applies to synapses that connect to dendrites of multicompartment model neurons. The main idea of this learning rule is to adjust the weights of dendritic synapses such that the dendrite can predict the firing rate of the soma. The dendrite expects the firing rate to be high when the dendrite's membrane potential is elevated due to many incoming spikes at the dendrite, and to be low if there are only a few incoming spikes. Thus, for this prediction to be true, synapses that transmit a spike toward the dendrite while the firing rate of the soma is low are depressed and those that provide input while the soma's firing rate is high are facilitated. Learning can be triggered by applying a teacher signal to the neuron via somatic synapses such that the actual somatic firing deviates from the dendritic prediction.

The plasticity rule is again of the general form (1), with a functional *F* on the right hand side that reads

(14) $$\begin{array}{l} F\left[s_{j}^{*},V_{i}^{*}\right]=\eta \;\kappa *\left(V_{i}^{*}\;s_{j}^{*}\right) \end{array}$$

(15) $$\begin{array}{l} \text{with }V_{i}^{*}=\left(s_{i}-\phi \left(V_{i}\right)\right)\;h\left(V_{i}\right),\\ \;s_{j}^{*}=\kappa_{s}*s_{j}. \end{array}$$

with exponential filter kernels κ and κ_*s*_ and non-linearities ϕ and *h*. Note that *F* depends on the postsynaptic spike train *s*_*i*_ via $V_{i}^{*}$. The latter can be interpreted as a prediction error, which never vanishes as spikes *s*_*i*_ (point process) are compared against a rate prediction ϕ(*V*_*i*_) (continuous signal).

In order to solve (1), we need to integrate over $F\left[s_{j}^{*},V_{i}^{*}\right]$, cf. (2). Writing down the convolution with κ explicitly, we obtain

(16) $$\begin{array}{l} \Delta W_{ij}\left(t,T\right)=\int_{t}^{T}dt^{\prime }\;F\left[s_{j}^{*},V_{i}^{*}\right]\left(t^{\prime }\right)\\ \;=\int_{t}^{T}dt^{\prime }\;\eta \int_{0}^{t^{\prime }}dt^{\prime \prime }\;\kappa \;\left(t^{\prime }-t^{\prime \prime }\right)\;V_{i}^{*}\left(t^{\prime \prime }\right)\;s_{j}^{*}\left(t^{\prime \prime }\right). \end{array}$$

A straight forward implementation of this expression is inefficient in terms of memory usage and computations because of the two nested integrals. However, since the kernels κ and κ_*s*_ are exponentials, one can perform one of the integrations analytically (see Appendix Section 5.1 for a derivation) to rewrite the weight change as

(17) $$\begin{array}{l} \Delta W_{ij}\left(t,T\right)=\eta \;[I_{1}\left(t,T\right)-I_{2}\left(t,T\right)+I_{2}\left(0,t\right)\left(1-e^{-\frac{T-t}{\tau_{\kappa }}}\right)], \end{array}$$

$$\begin{array}{l} \text{with }I_{1}\;(a,b)=\int_{a}^{b}dt\;V_{i}^{*}\;(t)\;s_{j}^{*}\;\left(t\right),\\ \;I_{2}\;(a,b)=\int_{a}^{b}dt\;e^{-\frac{b-t}{\tau_{\kappa }}}\;V_{i}^{*}\;\left(t\right)\;s_{j}^{*}\;\left(t\right), \end{array}$$

which is in line with the general formulation discussed in section 2.3.

## 3. Results

In the following, we first discuss time- and event-driven update schemes for synapses with voltage-based plasticity. Then we present a reference implementation for the Clopath rule (Clopath et al., 2010) and the Urbanczik-Senn rule (Urbanczik and Senn, 2014) in the spiking network simulator NEST (Jordan et al., 2019). Finally, we show that these implementations reproduce results of the original works and we assess their simulation performance on a distributed computing architecture.

### 3.1. Time-Driven vs. Event-Driven Update Scheme for Synapses With Voltage-Based Plasticity

**Figure 4:**
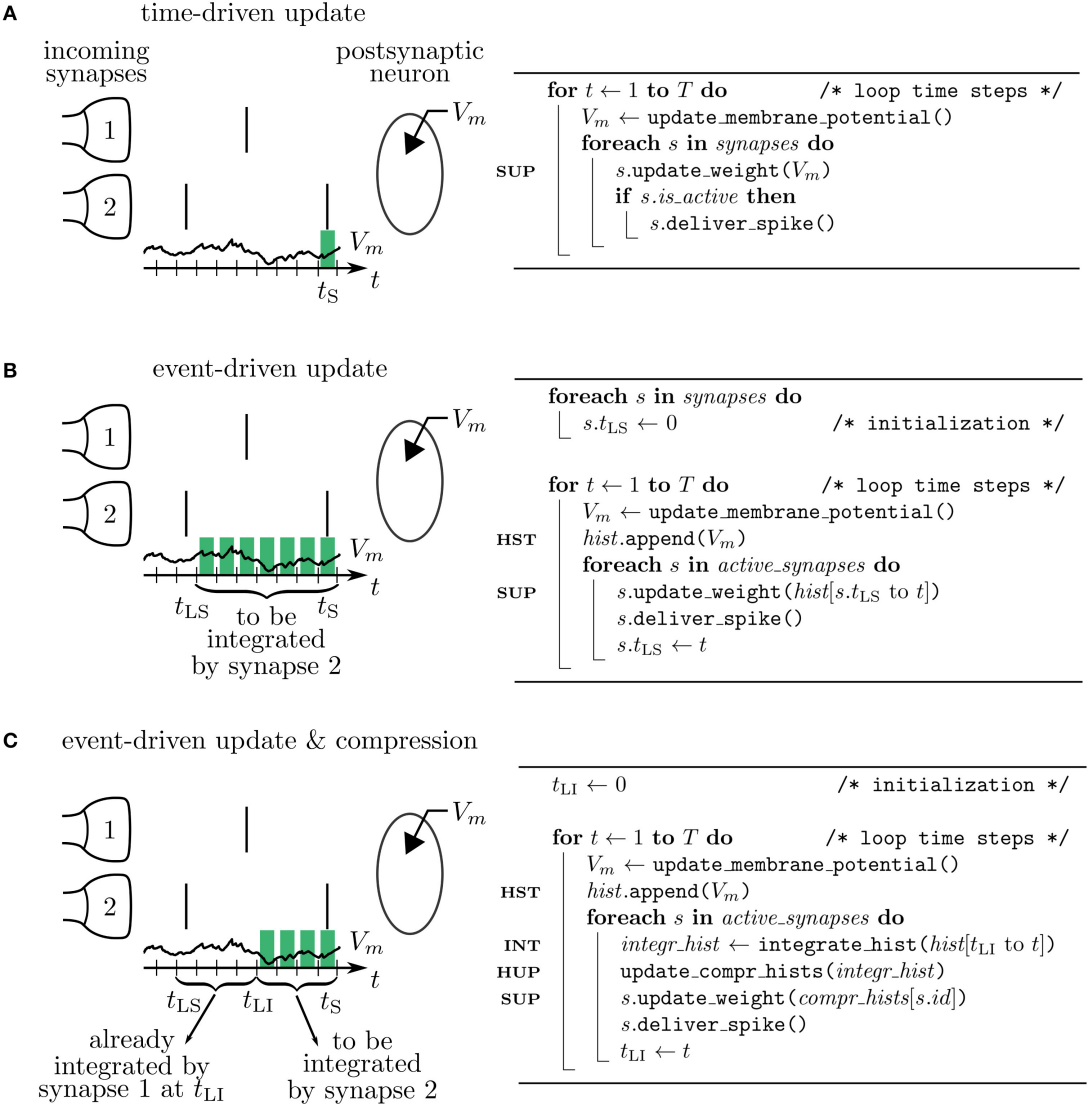
Simulation concepts. Left: illustration of processing the postsynaptic voltage trace *V*_*m*_ (*t*) for three simulation concepts. Two incoming synapses (1 and 2) transmit spikes (black, vertical bars) to the postsynaptic neuron. Depending on the algorithm, a different number of past membrane potentials has to be stored (green blocks) so that synapse 2 can update its weight when it delivers the spike at time *t*_*S*_. Right: corresponding pseudocodes. **(A)** In the time-driven update scheme the synaptic weight change is evaluated in every time step of the simulation for all the synapses. This requires only the latest value of the membrane potential to be accessible by the synapse to update its weight at *t*_*S*_ (see line marked SUP in pseudocode). **(B)** In the event-driven update scheme the computation of the synaptic weight change is performed only if a spike crosses the synapse. Therefore, storage of the time trace of *V*_*m*_ (see HST in code) from the last spike delivered by synapse 2 at *t*_LS_ up to the current time step *t*_*S*_ is needed. **(C)** In the compressed event-driven update scheme synapse 2 uses the time trace of *V*_*m*_ integrated from the last incoming spike at *t*_LI_ up to the current time step *t*_*S*_ (see INT in code) to complete its weight update (see SUP in code) and also to advance that of synapse 1. The preceding part of *V*_*m*_ from *t*_LS_ to *t*_LI_ was already integrated and applied to all incoming synapses (see HUP in code) by synapse 1 when it delivered the spike at *t*_LI_.

Let's assume in the following that *t*_LS_ and *t*_S_ denote two consecutive spike times of a presynaptic neuron *j*. The synaptic weight *W*_*ij*_(*t*_S_) corresponding to the spike at time *t*_S_ can be obtained from the weight *W*_*ij*_(*t*_LS_) at the time of the previous spike at *t*_LS_ and (6) by employing (8) to calculate the latter. As *F* mixes information of the pre- and postsynaptic neurons, this computation should be done in the synapse. Since there are no spikes in between *t*_LS_ and *t*_S_, it does not matter when the synapse is performing the updates of its weight. Two possibilities are: 1) Neurons calculate their own s^*^ and V^*^ for the current time step and make it accessible to the synapse to enable direct readout and update according to (8) in every time step. This method corresponds to a time-driven update of synapses (Figure 4A). 2) Neurons store a history of s^*^ and V^*^ and the synapse reads out this information at *t*_S_, i.e., at the time where the weight update becomes relevant for the network. This method corresponds to an event-driven update of synapses (Figure 4B). Both methods have their advantages and disadvantages analyzed in the following section.

#### 3.1.1. Time-Driven Scheme

**Figure 5:**
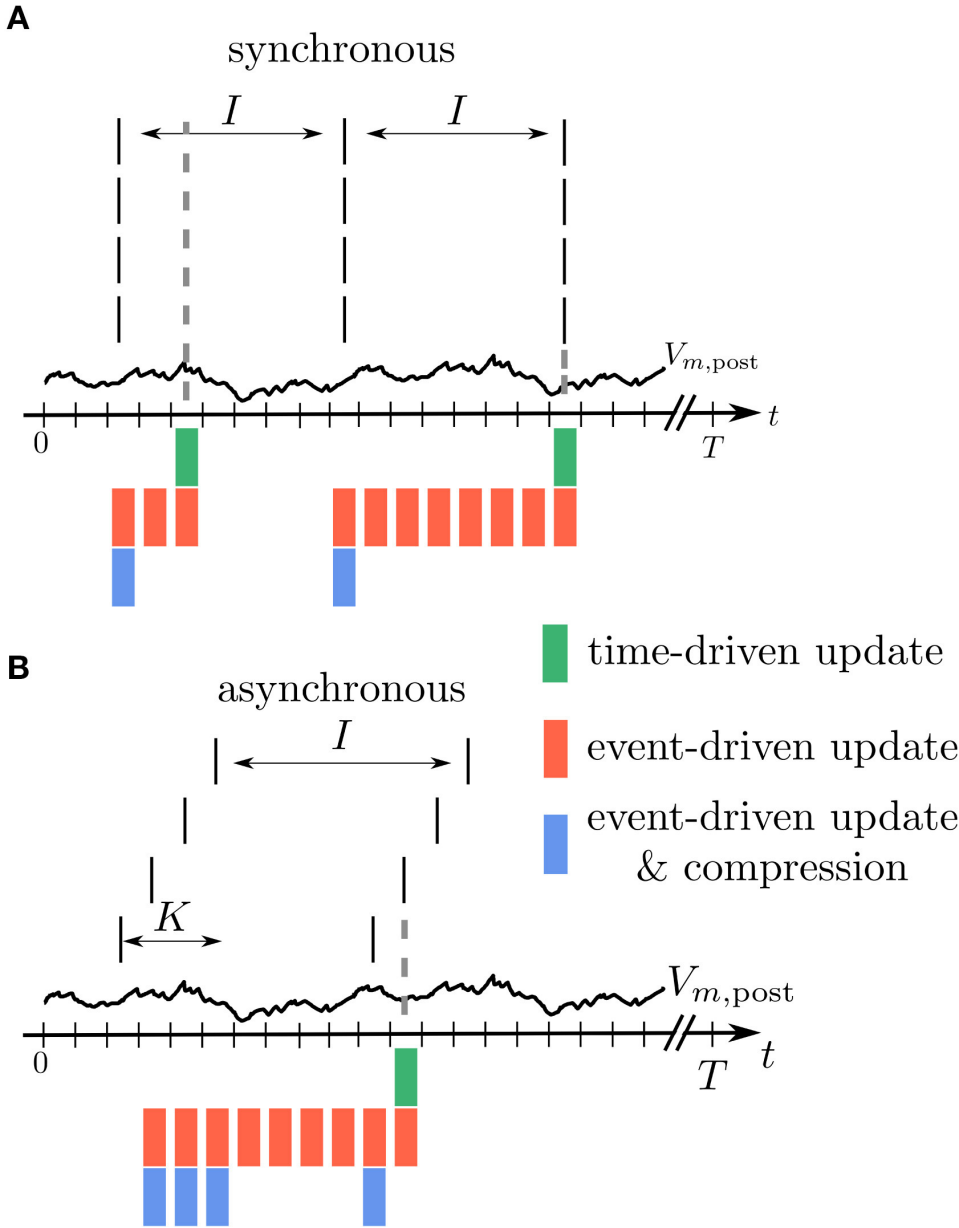
Illustration of buffer sizes for different simulation schemes in case of fully synchronous or asynchronous spikes. **(A)** All incoming spikes arrive synchronously: In the time-driven scheme the synaptic weight is updated in every time step of the simulation, so that only the current value of *V*_*m*,post_ needs to be available (green). In the event-driven scheme every synapse processes *V*_*m*,post_ from the last spike to the current one. Therefore, the relevant time trace needs to be stored (red). In the compressed event-driven scheme this part of *V*_*m*,post_ is processed only once and used to update the weight of all the synapses. Since the weight change is a function of the last spike time which is the same for all the synapses, only one value needs to be updated (blue). In this situation the length *L* of the compressed history is *i* = 1, see Table 1. **(B)** All incoming spikes arrive in different time bins: For the time-driven and the event-driven scheme the scenario is similar to **(A)**. For the compressed event-driven scheme the number of values that need to be updated equals the number of incoming synapses *K* so that *i* = *K*.

In a time-driven update scheme the information on the membrane potential is directly processed by the synapses such that only the current value of the membrane potential needs to be stored, corresponding to a membrane potential history of length *L* = 1 (Figure 5 and Table 1). For a simulation of *T* time steps, the history needs to be manipulated *H* = *T* times: the single stored value gets updated once per time step. The price that comes with the short history is that synapses need to be updated as often as neurons. This amounts to *M* = *K* · *T* function calls to synapse code for each neuron. Here *K* denotes the in-degree of each neuron. Each function call of synapse code causes a single computation of $\Delta W_{ij}\left(t^{\alpha },t^{\alpha +1}\right)$, giving rise to in total *C* = *K* · *T* computations per neuron. The membrane potential trace is thus effectively integrated *K* times; once for each synapse. As both *K* and *T* are large numbers in typical simulations of plastic cortical networks, the amount of function calls and computations is therefore large in this setting. The time-driven scheme furthermore forces the execution of synapse code also at time steps where no update would be required, i.e., at time steps, where $s_{i}^{*},s_{j}^{*},V_{i}^{*}$ have values for which $\Delta W_{ij}\left(t^{\alpha },t^{\alpha +1}\right)=0$. In addition, for delayed connections a history of $V_{i}^{*}$ of length *L* = *d*_max_ of the maximal delay *d*_max_ measured in simulation steps needs to be stored. We here assume the delay to be on the postsynaptic side; it represents the time the fluctuations of the somatic membrane potential propagate back through the dendrites to the synapses. Therefore, *F* does not depend on $V_{i}^{*}\left(t\right)$, but on $V_{i}^{*}\left(t-d_{j}\right)$ with a delay *d*_*j*_ encoding the location of the synapse with presynaptic neuron *j*.

**Table 1:** Comparison of synapse update schemes.

	**Time-driven**	**Event-driven**	**Event-driven & compression**
History length *L*	1	*I*	*i*
Synapse function calls *M*	*K* · *T*	*K* · *T*/*I*	*K* · *T*/*I*
Weight change computations *C*	*K* · *T*	*K* · *T*	*T*
History entry manipulations *H*	*T*	*T*	*K* · *T*/*I* · *i*

*From the view point of a postsynaptic neuron, the table shows the maximal length of the history L, the number of function calls M of synapse code, the number of computations C of infinitesimal weight changes $\Delta W_{ij}\left(t^{\alpha },t^{\alpha +1}\right)$, and the number of history entry manipulations H for a simulation of T time steps, a uniform inter-spike interval I between spikes of a single presynaptic neuron, and an in-degree K for each neuron and no delays. For the event-driven compression scheme the entries show the length of the compressed history where i is the number of different spike times within the last inter-spike interval I*.

#### 3.1.2. Event-Driven Scheme

In an event-driven update scheme for synapses, the time trace of the membrane potential $V_{i}^{*}$ needs to be stored until all incoming synapses have read out the information to update their weight for a given period. The storage and management of such a history can be expensive in terms of memory and runtime. In each time step, the value of the current membrane potential is appended to the history, leading to *H* = *T* history manipulations for a simulation of *T* time steps. Assuming for simplicity a homogeneous inter-spike interval of *I* time steps between consecutive spikes of single neurons, we in the following showcase some qualitative history sizes. As synapses need all values of $V_{i}^{*}$ in between two consecutive spikes, the maximum history length is *L* = *I* (Figure 5). In case of different firing rates, *I* corresponds to the maximum inter-spike interval of any of the presynaptic neurons. Synapse code in this scheme is, however, only called in the event of a spike, leading to only *M* = *K* · *T*/*I* function calls per neuron, where *T*/*I* is the number of spikes passing a single synapse during the simulation of *T* time steps. The total amount of computations *C* of weight changes $\Delta W_{ij}\left(t^{\alpha },t^{\alpha +1}\right)$ is of course unchanged with respect to the time-driven scheme; they are just split across fewer function calls (*C* = *M* · *L* = *K* · *T*). Table 1 immediately shows the trade-off between memory consumption (length of history) and run time (number of function calls): the event-based scheme consumes more memory, but is faster than the time-driven scheme. Note that since a history of the membrane potential is stored anyway, this scheme is naturally applicable to connections with different delays. A further performance increase can be achieved in plasticity rules, where weight changes only happen under certain conditions on $V_{i}^{*}$: if values $\Delta W_{ij}\left(t^{\alpha },t^{\alpha +1}\right)\neq 0$ are rare, a non-continuous history can be stored. In such a scenario, time stamps need to be stored alongside the membrane potential to enable synapses to read out the correct time intervals (see section 3.2.3).

#### 3.1.3. Event-Driven Compression

The event-driven compression scheme is a modified event-driven scheme that makes use of the fact that for a specific class of plasticity rules the integrated time trace of the membrane potential $V_{i}^{*}$ can be used to advance the weight update of all incoming synapses, see section 2.3.1. Therefore, the time trace of $V_{i}^{*}$ stored in the postsynaptic neuron only needs to extend back to the last incoming spike (denoted by *t*_LI_ in Figure 4C). This way the history of $V_{i}^{*}$ is always short, as the total rate of incoming spikes is high in physiological network states. Due to the dependence of the weight update on the time of the last spike that crossed the synapse, the postsynaptic neuron stores the compressed history of length *L* = *i*, where *i* is the number of different spike times within the last inter-spike interval *I* (Figure 5). The compressed history is consequently never larger than the history length *L* = *I* of the ordinary event-driven scheme (Figure 5B). For synchronous spikes where the last presynaptic spike time is the same for all synapses, the compressed history, however, contains only one entry (Figure 5A). Still, synapse code is executed at every spike event, giving rise to *M* = *K* · *T*/*I* function calls. The full membrane potential trace of length *T* is effectively only integrated once, amounting to in total *C* = *T* infinitesimal weight change computations that are performed in batches in between any two incoming spike events (Table 1). The price for this is that history updates are more expensive: instead of appending a single entry in each time step, at each spike event the full compressed history is updated, giving rise to in total *H* = *M* · *i* = *K* · *T* · *i*/*I* history entry manipulations, as opposed to *H* = *T* in the time- and ordinary event-driven schemes (Table 1). In practice, infinitesimal weight change computations are, however, often more costly than history updates, such that the compression algorithm achieves a performance increase (see section 3.4).

Finally, a drawback of the event-driven compression is that it relies on the fact that all synapses use the same processed membrane potential $V_{i}^{*}$. For distributed delays, Δ*W*_*i*_(*t*_*LS*_, *T*) has a dependence on the presynaptic neuron *j* via $V_{i}^{*}\left(t-d_{j}\right)$. In this case, a separate compressed history needs to be stored for every different delay of connections to the neuron.

### 3.2. Reference Implementation in Network Simulator With Event-Based Synapse Updates

This section describes the implementation of two example voltage-based plasticity rules by Clopath et al. (2010) and Urbanczik and Senn (2014) in a spiking neural network simulator that employs a time-driven update of neurons and an event-based update of synapses. While the naming conventions refer to our reference implementation in the simulation software NEST, the algorithms and concepts presented below are portable to other parallel spiking network simulators.

The Clopath and Urbanczik-Senn rule are chosen as widely used prototypical models of voltage-based plasticity. The differences in the two rules help to exemplify the advantages and disadvantages of the algorithms discussed in section 3.1. As originally proposed, they are implemented here for two different types of neuron models, Adex and Hodgkin-Huxley point-neurons for the Clopath rule (aeif_psc_delta_clopath and hh_psc_alpha_clopath) and two-compartment Poisson neurons (pp_cond_exp_mc_urbanczik) for the Urbanczik-Senn rule. Extensions to multiple dendritic compartments in the latter case are straight forward. Our implementation of aeif_psc_delta_clopath follows the reference implementation on ModelDB which introduced a clamping of the membrane potential after crossing the spiking threshold to mimic an action potential. Details can be found in Appendix Section 5.2.

The plasticity rules differ in the state variable that is being stored and its interpretation. For the Clopath rule, the stored variable is a thresholded and filtered version of the membrane potential that takes into account characteristics of membrane potential evolution within cells in the vicinity of spike events. The restriction to temporal periods around spikes suggests to implement a history that is non-continuous in time. In contrast, the Urbanczik-Senn rule uses the dendritic membrane potential to predict the somatic spiking; the resulting difference is taken as an error signal that drives learning. This error signal never vanishes and thus needs to be stored in a time-continuous history.

Finally, the proposed infrastructure for storing both continuous and non-continuous histories is generic so that it can also be used and extended to store other types of signals such as external teacher signals.

#### 3.2.1. Exchange of Information Between Neurons and Synapses

The implementation of voltage-based plasticity rules in NEST follows the modular structure of NEST, key part of which is the separation between neuron and synapse models. This separation makes it easy for a newly added neuron model to be compatible with existing synapse models and vice versa. A downside is that information, such as values of parameters and state variables, is encapsulated within the respective objects. Simulations in NEST employ a hybrid parallelization scheme: OpenMP threads are used for intra node parallelization and the Message Passing Interface (MPI) for inter node communication. In parallel simulations, synapses are located at the same MPI process as the postsynaptic neurons (Morrison et al., 2005). Thereby, no communication between MPI processes is needed for the required transfer of information between postsynaptic neurons and synapses to compute weight changes of connections and only one spike needs to be communicated by a given source neuron for all target neurons living on the same MPI process.

The model of STDP requires synapses to access spike times of postsynaptic neurons. In order to provide a standardized transfer of this information across all neuron models that support STDP, in recent years the so-called Archiving_Node has been introduced as a parent class of the respective neuron models (Morrison et al., 2007a). It provides member functions to store and access spike histories. If a neuron model supports STDP, it only needs to be a child of Archiving_Node and contain one additional line of code, namely a call of the function set_spiketime(), which stores the time of outgoing spike events. We here extended this framework for voltage-based plasticity rules and enhanced the functionality of the archiving node by the member functions write_history(), get_history(), and compress_history() to additionally store, read out and manipulate voltage traces or other continuous signals (for Details, see Appendix Section 5.3). To avoid overhead for simulations with only STDP synapses, we introduced two child classes of Archiving_Node, Clopath_Archiving_Node, and Urbanczik_Archiving_Node, that each provide containers and functions for the specific histories required for the two plasticity rules. Neuron models that support the respective synapse model then derive from the child classes instead of the root level archiving node.

#### 3.2.2. Delays and Min_delay Communication

All synapses implemented in NEST are so far purely event-driven. To assess the performance of the time-driven update scheme of synapses with voltage-based plasticity, we also implemented a time-driven version of the Clopath and Urbanczik-Senn synapse. Spiking network simulators exploit the delay of connections to reduce communication between compute processes (Morrison et al., 2005): Instead of sending each spike individually, spikes are buffered and sent in a batch after a certain period. The length of this period, the min_delay, corresponds to the minimal delay of all connections in the network. The buffering of spikes within this period is possible because the earliest time point that a spike at time *t*_S_ can affect the postsynaptic membrane potential is at *t* = *t*_S_ + min_delay. In between *t*_S_ and *t* neurons are decoupled such that their state variables can be propagated forward in time independent of each other and in a batch (Morrison and Diesmann, 2008). We implemented the same min_delay update scheme for synapses, by imposing a function call to time-driven synapses in every min_delay period to update their synaptic weight. If min_delay equals the simulation step size *h*, this scheme corresponds to the scheme explained in section 3.1.1. Making use of the min_delay infrastructure of NEST speeds up simulations with time-driven synapses in the case *d* > *h* as fewer function calls to synapses are needed (see section 3.4). In case of simulations with synaptic delays, the time-driven update scheme requires the storage of a history of the membrane potential of length max_delay.

Storing state variables in event-driven schemes is more complex as the history does not have a fixed length max_delay. Instead it needs to be dynamically extended and shortened. A long history can occupy a large amount of memory and its processing by the synapses becomes computationally expensive. Therefore, it is advantageous to optimize the way how information is stored and accessed and how entries that are no longer needed can be identified for deletion. For details of these optimizations in our NEST implementation, see Appendix Section 5.3.

As discussed in section 3.1.3, the event-based compression scheme relies on the fact that all synapses to one postsynaptic neuron employ the same $V_{i}^{*}$. This is not the case if delays of the corresponding connections are distributed. The compression scheme can therefore only be efficient if all delays have a fixed value. If spikes are processed and synapses are updated in a chronological order, then a well-defined segment of the history of $V_{i}^{*}$ can be integrated and the compressed history can be updated. In NEST, spikes are, however, buffered within a period of min_delay before being sent and processed. Consequently, synapses are not necessarily updated in chronological order. Therefore, the event-based compression scheme can only be implemented in NEST in the case where delays equal the simulation time step. Future work may explore whether the latter restriction could be overcome by sorting all incoming spike events of a given postsynaptic neuron prior to delivery.

#### 3.2.3. Specifics of Clopath Plasticity

We implement both an adaptive exponential integrate-and-fire neuron model (aeif_psc_delta_clopath) and a Hodgkin-Huxley neuron model (hh_psc_alpha_clopath) supporting Clopath plasticity. These implementations consider the filtered versions ū_±_ of the membrane potential as additional state variables of the neuron. Thereby, they can be included in the differential equation solver of the neurons to compute their temporal evolution. Parameters of κ_±_ consequently need to be parameters of the neuron object rather than the synapse. The same is true for the values of θ_±_; they are used in the neuron to determine whether $V_{i,\text{LTP}}^{*}$ and $V_{i,\text{LTD}}^{*}$ evaluate to zero, which systematically happens due to the Heaviside functions in their definitions.

The LTD mechanism is convenient to implement within the event-driven framework: when the synapse is updated at time *t*, it reads the values ū_−_ (*t* − *d*) and θ_−_ from its target and computes the new weight. Here, *d* denotes the dendritic delay of the connection that accounts for the time it takes to propagate somatic membrane potential fluctuations to the synapse. The archiving node contains a cyclic buffer, also called ring buffer, that stores the history of ū_−_ for the past max_delay time steps so that the synapse can access a past value of this quantity. Consequently, the LTD history is always short and can be forgotten in a deterministic fashion.

The computation of the weight change due to LTP requires the evaluation of the integral over $V_{i,\text{LTP}}^{*}\left(t\right)$. The latter is stored in the archiving node as a vector whose elements are objects that contain three values: the corresponding time *t*, the value of $V_{i,\text{LTP}}^{*}$ and an access counter that initially is set to zero.

##### 3.2.3.1. Time-Driven Update

For simulations with homogeneous delays equal to the simulation time step, the history of $V_{i,\text{LTP}}^{*}$ always contains only a single value as it is read out in every time step by all synapses. For larger delays, the history is of length max_delay, and each synapse reads out a segment of length min_delay, increasing the access counter of the corresponding entries by one. For the last synapse that requests a certain segment, the access counter then equals the in-degree *K*, which is the criterion to delete the corresponding entries from the history. Although for simplicity done in our reference implementation, the time-driven scheme does not require us to store the time stamp *t* of each history entry. The overhead of this additional number is, however, negligible.

##### 3.2.3.2. Event-Driven Update

In event-driven schemes, the history of $V_{i,\text{LTP}}^{*}$ dynamically grows and shrinks depending on the spikes of presynaptic neurons. Since many values of $V_{i,\text{LTP}}^{*}$ are zero, it is beneficial to only store the non-zero values. In this case, a time stamp of each entry is required to assign values of the non-continuous history of $V_{i,\text{LTP}}^{*}$ to their correct times. In case of the uncompressed scheme, when a synapse *j* is updated at time *t*_*S*_ of a spike, it requests the part of the history between the last spike *t*_*LS*_ and the current spike *t*_*S*_ (minus the dendritic delay, see Appendix Section 5.3) from the archiving node. This history segment is then integrated in synapse *j* and used for its weight update. Each synapse thus integrates the history $V_{i,\text{LTP}}^{*}$ anew (section 3.1.2). For the compressed scheme, the history of $V_{i,\text{LTP}}^{*}$ is integrated between the last incoming spike at *t*_LI_ and the current spike at *t*_S_ inside the archiving node. Using this newly integrated time trace, the weight of synapse *j* is updated and the compressed history for all other last spike times is advanced. Afterwards the history of $V_{i,\text{LTP}}^{*}$ is deleted. Thereby, $V_{i,\text{LTP}}^{*}$ is only integrated once for all synapses.

In any case, the integrated history of $V_{i,\text{LTP}}^{*}$ needs to be combined with the presynaptic spike trace $s_{j}^{*}$. The latter is easily computed analytically inside the synapse because it is an exponential decay of the corresponding value at the time of the last spike. At the end of the update process the trace is increased by $\tau_{s}^{-1}$ to account for the trace of the current spike, where τ_*s*_ is the time constant of the kernel κ_*s*_.

#### 3.2.4. Specifics of Urbanczik-Senn Plasticity

**Figure 6:**
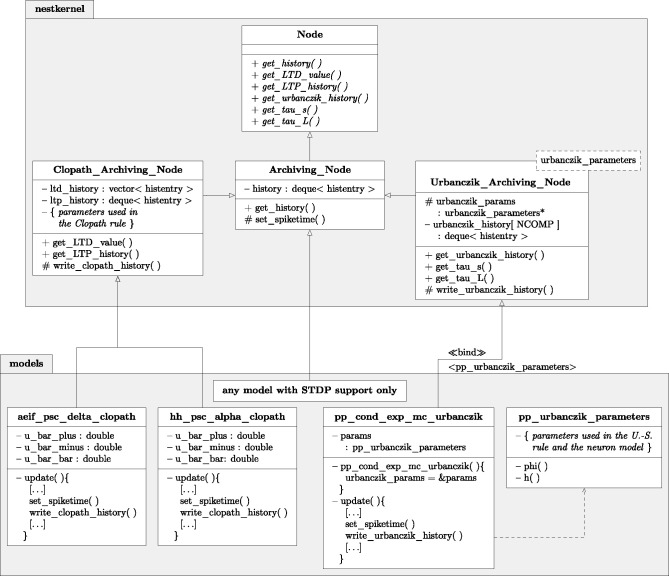
Class diagram of NEST classes and functions. Simplified class diagram for embedding the Clopath **(Left)** and Urbanczik-Senn rule **(Right)** in the NEST infrastructure. The code is distributed across the nestkernel and neuron models. nestkernel contains the base class Node of all neurons models. Models that support ordinary STDP are derived from the Archiving_Node, models that can use the Clopath synapse (aeif_psc_delta_clopath and hh_psc_alpha_clopath) or Urbanczik-Senn synapse (pp_cond_exp_mc_urbanczik) are derived from the Clopath_Archiving_Node or the Urbanczik_Archiving_Node, respectively. The latter add the required functions for storing and managing the history of continuous quantities. The model pp_cond_exp_mc_urbanczik requires a helping class pp_urbanczik_parameters because the Urbanczik_Archiving_Node needs to access functions and parameters that are specific to the neuron model and therefore not located in the Urbanczik_Archiving_Node to keep its implementation more general.

Following the original publication (Urbanczik and Senn, 2014), we implement a Poisson spiking neuron model (pp_cond_exp_mc_urbanczik) supporting Urbanczik-Senn plasticity. One peculiarity of this model is that the gain function ϕ that translates the membrane potential into a firing rate also enters the plasticity rule through V^*^. Therefore ϕ as well as its parameters need to be known by the neuron and the synapse. Creating an additional helper class (pp_urbanczik_parameters) as a template argument for the corresponding archiving node (Urbanczik_Archiving_Node) and neuron model (pp_cond_exp_mc_urbanczik) solves this problem (Figure 6): it contains all parameters and functions required by both classes. As explained in section 2.5, the representation (17) is more beneficial for implementing the Urbanczik-Senn rule than that of (16). The first two integrals in (17) only extend from *t* to *T*; history entries for times smaller than *t* are not needed and can be deleted after the corresponding update. The dependence on the full history back until 0 arising from the convolution with κ is accumulated in the last term in (17), which the synapse computes with the help of storing one additional value *I*_2_(0, *t*). At the end of a weight update this value is overwritten by the new value $I_{2}\left(0,T\right)=e^{-\frac{T-t}{\tau_{\kappa }}}I_{2}\left(0,t\right)+I_{2}\left(t,T\right)$ which is then used in the next update. Either the synapse (time- and event-driven update) or the archiving node (event-driven compression) compute the two integrals *I*_1_ and *I*_2_ but in all cases the archiving node stores the history of $V_{i}^{*}\;(t)$.

### 3.3. Reproduction of Results in Literature

**Figure 7:**
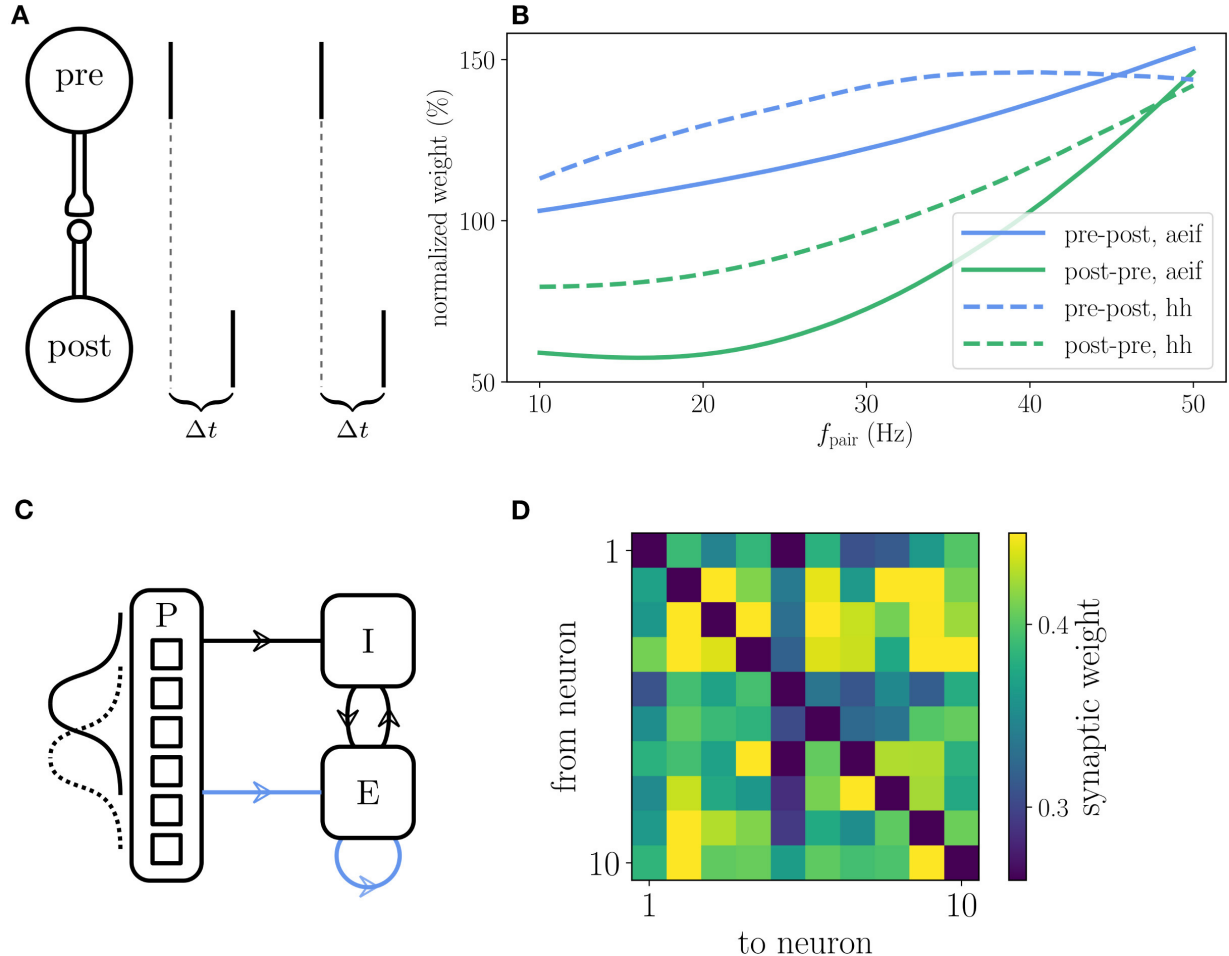
Reproduction of results with Clopath rule. **(A)** Setup of the spike pairing experiment. Two neurons (“pre” and “post”) that are connected by a plastic synapse receive input so that they spike one after another with a delay Δ*t*. The change of the synaptic weight is computed according to the Clopath rule as a function of the frequency *f*_pair_ with which the spike pairs are induced. **(B)** Result of the spike pairing experiment. The relative change of the synaptic weight after five spike pairs as a function of *f*_pair_ is shown for two different neuron models (aeif: solid curves, Hodgkin-Huxley: dashed curves). The blue curves represent a setup where the postsynaptic neuron fires after the presynaptic one (pre-post, Δ*t* = 10 ms) and the green curves represent the opposite case (post-pre, Δ*t* = −10 ms). This panel corresponds to Figure 2B of Clopath et al. (2010). **(C)** Setup of the network that produces strong bidirectional couplings. The network consists of an inhibitory (I) and an excitatory (E) population which receive Poisson spike trains (P) as an external input. The firing rate of the latter is modulated with a Gaussian shape whose center is shifted every 100 ms. The external input connections to the excitatory population are plastic as well as the connections within the excitatory population (indicated by blue arrows). **(D)** Synaptic weights of the all-to-all connected excitatory neurons after the simulation of the network. Strong bidirectional couplings can be found, e.g., between neurons 2 and 3, 2 and 9, and 4 and 7. The setup of this experiment is similar to that shown in Figure 5 of Clopath et al. (2010). A more detailed description of the two experiments can be found in Appendix Section 5.4.

The reference implementation of the Clopath plasticity reproduces the results from Clopath et al. (2010) on the frequency dependence of weight changes in spike-pairing experiments and the emergence of bidirectional connections in small all-to-all connected networks (Figure 7). The setup of the spike-pairing experiment in Figure 7A consists of two neurons connected via a plastic synapse. The pre- and postsynaptic neuron are forced to spike with a time delay of Δ*t* multiple times which leads to a change in synaptic weight that depends on the frequency of the spike pairs (Figure 7B). The setup of the small network is shown in Figure 7C. The weights of the plastic synapses within the recurrently connected excitatory population are initialized all to the same value. At the end of the simulation during which the network receives a time varying input, some pairs of neurons show strong bidirectional connections (Figure 7D). See Appendix Section 5.4 for details on the setup of both experiments as implemented in NEST.

**Figure 8:**
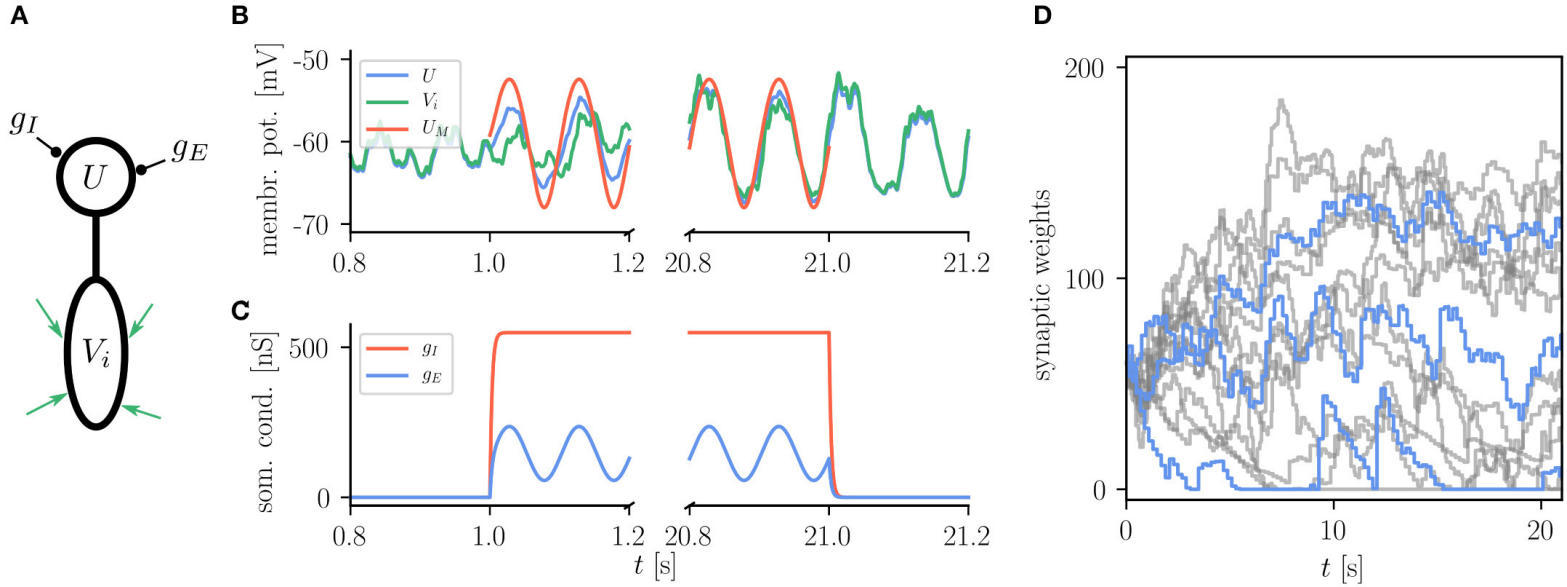
Reproduction of results with Urbanczik-Senn rule. **(A)** Setup of a simple learning task using the Urbanczik-Senn plasticity rule. The somatic conductances *g*_*I*_ and *g*_*E*_ of a two-compartment neuron are modulated such that they induce a teaching signal with sinusoidal shape. The dendrite receives a repeating spike pattern as an input via plastic synapses (green arrows). **(B)** The synapses adapt their weights so that the somatic membrane potential *U* (blue) and the dendritic prediction *V*_*i*_ (green) follow the matching potential *U*_*M*_ (red) after learning. **(C)** Excitatory (*g*_*E*_) and inhibitory (*g*_*I*_) somatic conductances that produce the teaching signal. **(A,B)** correspond to Figure 1 of Urbanczik and Senn (2014). **(D)** Temporal evolution of the synaptic weights during learning. For the sake of better overview, only a subset of weights is shown (gray) with three randomly chosen time traces highlighted in blue. Synapses in NEST fulfill Dale's principle which means that a weight update cannot convert an excitatory into an inhibitory synapse and vice versa giving rise to the rectification at zero.

The basic use of the Urbanczik-Senn rule in NEST is exemplified in Figure 8 which shows the reproduction of a simple learning experiment from the original publication (Urbanczik and Senn, 2014). Here the neuron is supposed to transform spike patterns in the input to the dendritic compartment into a sinusoidal modulation of the somatic membrane potential. This target potential is determined by an external teaching signal during learning. Via minimizing the error between the dendritic prediction of the somatic membrane potential and the actual somatic membrane potential, weights of dendritic synapses are organizing such that the neuron can produce the desired membrane potential. There is, however, no stop-learning region in the Urbanczik-Senn rule (for a modified version, see Cartiglia et al., 2020): The error never vanishes completely which causes weights to keep changing despite an overall good approximation of the target signal. Details of the experiment and NEST setup can be found in Appendix Section 5.6.

### 3.4. Performance of the Reference Implementations

#### 3.4.1. Clopath Plasticity

In order to evaluate the performance of the implementation of the Clopath rule in NEST, in a weak-scaling setup, we simulate excitatory-inhibitory networks of increasing size, but fixed in-degree *K*. As we expect the performance to critically depend on the number of synapses, we examine two scenarios: a small in-degree *K* = 100, and a rather large in-degree *K* = 5, 000. While the first case might be suitable for small functional networks, the latter in-degree represents a typical number for cortical networks. Further details on network and simulation parameters are given in Supplementary Table 5. As a reference, we also simulate the same network with STDP synapses, which require much fewer computations as they rely solely on spike times. To achieve the same network state, that is the same spikes, for the different connectivity rules, we impose the weights to stay constant across time by setting learning rates to zero. This way all computations for weight changes are being performed, but just not applied. This has the additional advantage that reasonable asynchronous irregular network states are simple to find based on predictions for static synapses (Brunel, 2000).

**Figure 9:**
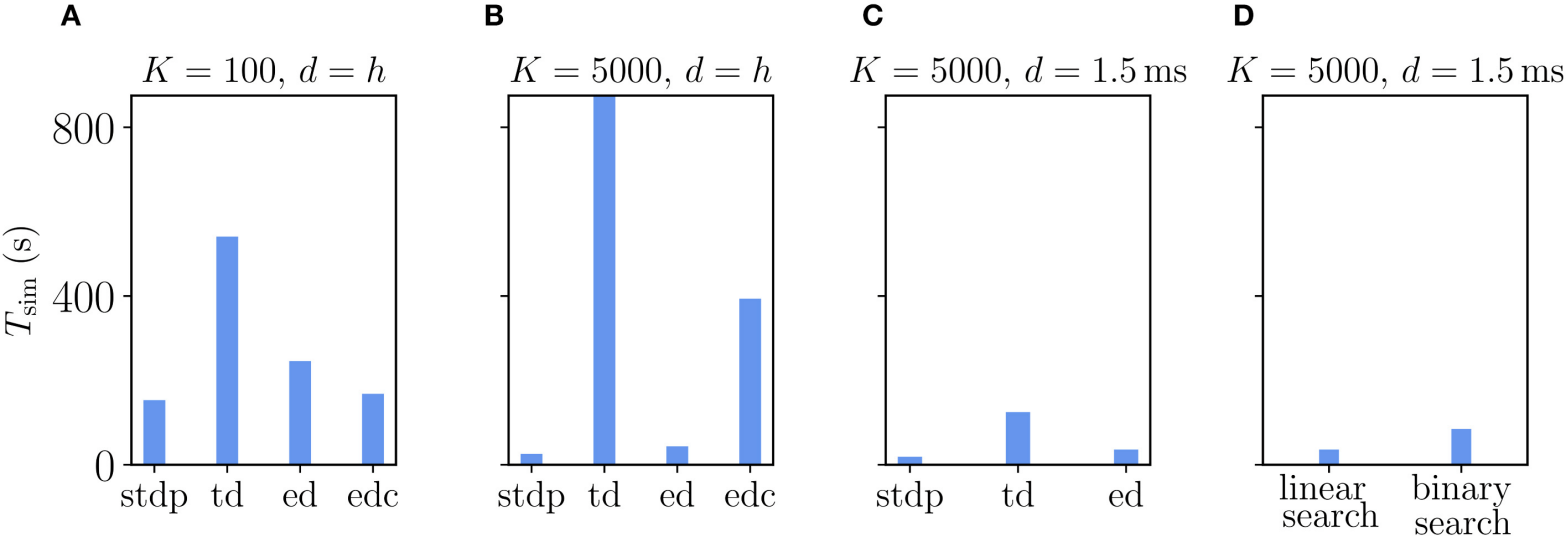
Comparison of simulation times *T*_sim_ for excitatory-inhibitory networks with different implementations of the Clopath plasticity in NEST. Simulation times exclude network building and only account for updates of the dynamical state of the system. The following implementations are shown: “stdp”: standard implementation of STDP synapse, “td”: time-driven implementation of Clopath synapse, “ed”: event-driven scheme as included in NEST 2.20.1, “edc”: event-driven compression. **(A)** Network of size *N* = 1.92 · 10^6^ with small in-degree *K* = 100 and all synapses having a delay *d* equal to the resolution of the simulation *h* = 0.1 ms. **(B)** Network of size *N* = 1.54 · 10^5^ with large in-degree *K* = 5, 000 and *d* = *h*. **(C)** Same network as in **(B)** but *d* = 1.5 ms (for *d* > *h* “edc” not compatible with NEST, see section 3.1.3). In **(A–C)** both “ed” and “edc” use linear search of the history and access counters, see Appendix Section 5.3. **(D)** Comparison between “ed”-implementations using linear search and direct computation of the position, see Appendix Section 5.3.0.2. All simulations use 768 threads distributed over 32 compute nodes each running one MPI process. Further parameters as in Supplementary Table 5.

The Clopath rule has originally been proposed for connections without delays (Clopath et al., 2010). Therefore, we first evaluate its performance in this setting (delay equals simulation time step), which is, however, not the natural setting for a simulator like NEST that makes use of delays to speed up communication between compute processes. The first observation is that, as expected, simulations with Clopath synapses are slower than those with ordinary STDP (Figure 9). Given the update of synapses in every simulation step, the time-driven scheme for Clopath synapses is much slower than the event-driven scheme (Figure 9A). The difference becomes larger the more synapses there are (Figure 9B). Introducing a delay leads to fewer function calls to synapses (once every min_delay) and therefore increases the speed of the time-driven scheme (Figure 9C). Its simulation times, however, remain much above the event-driven scheme. This comparison illustrates the benefit of event-driven updates for Clopath synapses.

**Figure 10:**
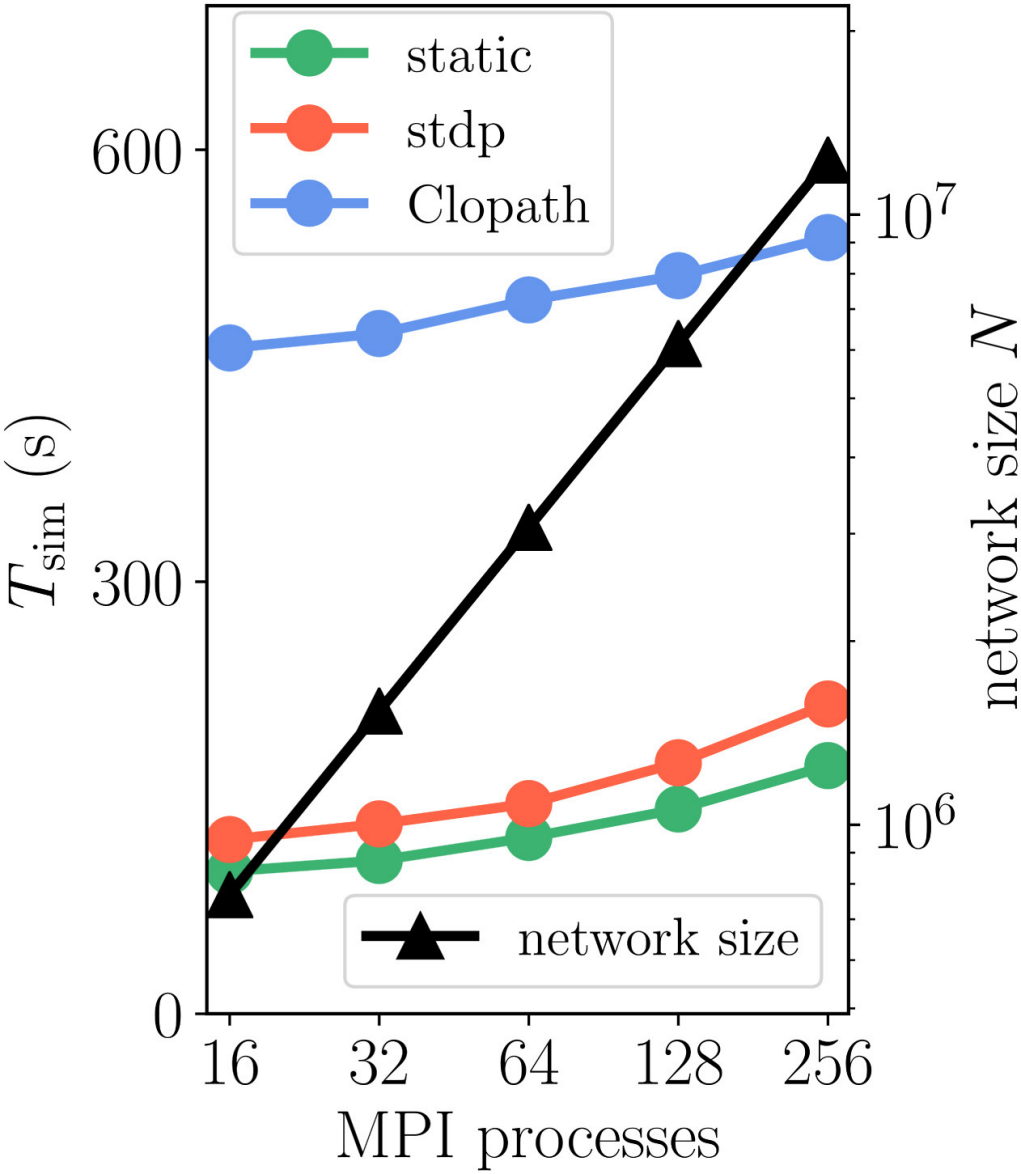
Scaling of simulation time *T*_sim_ with network size for 2 *s* of biological time: Clopath plasticity. Weak scaling: computational resources (horizontal axis) increase proportionally to network size *N* (black curve and triangles, right vertical axis). Event-driven scheme for Clopath rule (blue) compared to static (green) and STDP synapse (red). Network and simulation parameters as in Supplementary Table 5 with in-degree *K* = 5, 000. For all simulations each compute node runs one MPI process with 24 threads.

How does compression of the history change the picture? As discussed in section 3.1.3, compression has the advantage of not integrating the membrane potential history for each synapse separately. A downside of the event-based compression is that it requires storing one history entry for each last spike time of presynaptic neurons. For large in-degrees, this history is therefore longer than the history of $V_{i,\text{LTP}}^{*}$, which we implemented as non-continuous for the Clopath rule. Consequently, the event-based compression scheme only outperforms the ordinary event-driven scheme for small in-degrees (Figure 9A), but not for large in-degrees (Figure 9B). Given that the compression can only be implemented in NEST for connections with delay equal to the resolution of the simulation (see section 3.2.2), the method of choice is therefore the ordinary event-driven scheme (section 3.1.2). Although a bit slower, its run-time is on the same order of magnitude as the ordinary STDP synapse, with similar weak-scaling behavior (Figure 10). The additional computations with respect to STDP result in a constant overhead.

Another advantage of having short non-continuous histories is that searching the history at readout is fast. A simple linear iteration scheme is therefore even faster than a binary search (Figure 9D) because the latter search requires an additional list of presynaptic spike times (see Appendix Section 5.3) which is unnecessary overhead in this scenario. As a result the ordinary event-driven scheme with linear history iteration is the most general and efficient scheme and therefore integrated into NEST 2.20.1 (Jordan et al., 2019).

#### 3.4.2. Urbanczik-Senn Plasticity

**Figure 11:**
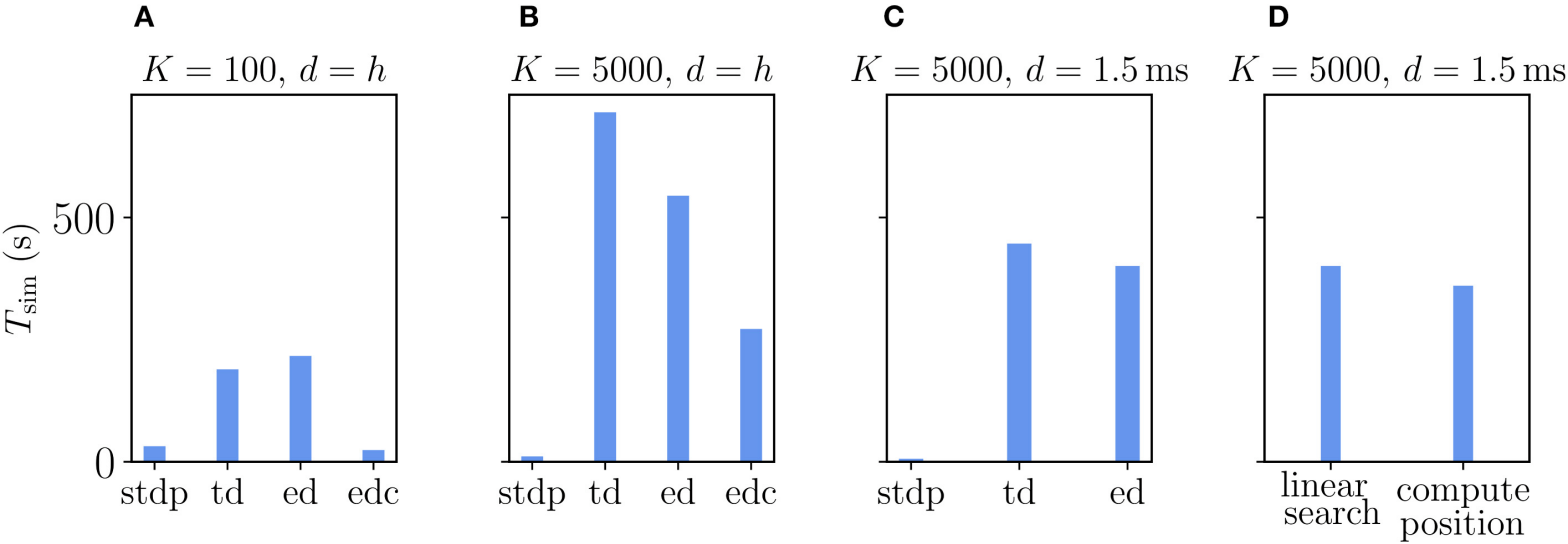
Comparison of simulation times *T*_sim_ for excitatory-inhibitory networks with different implementations of the Urbanczik-Senn plasticity in NEST. The following implementations are shown: “stdp”: standard implementation of STDP synapse in NEST, “td”: time-driven implementation of Urbanczik-Senn synapse, “ed”: event-driven scheme, edc”: event-driven compression. **(A)** Network of size *N* = 3.84 · 10^5^ with small in-degree *K* = 100 and all synapses having a delay *d* equal to the resolution of the simulation *h* = 0.1 ms. **(B)** Network of size *N* = 3.84 · 10^4^ with large in-degree *K* = 5, 000 and *d* = *h*. **(C)** Same network as in **(B)** but *d* = 1.5 ms (for *d* > *h* “edc” not compatible with NEST, see section 3.1.3). In **(A–C)** both “ed” and “edc” use linear search of the history and the access counters, see Appendix Section 5.3. **(D)** Comparison between “ed”-implementations using linear search and direct computation of the position, see Appendix Section 5.3.0.2. All simulations use 768 threads distributed over 32 compute nodes each running one MPI process. Details on network parameters in Supplementary Table 5.

The Urbanczik-Senn rule, in its original version, does not account for delays in connections (Urbanczik and Senn, 2014). As for the Clopath rule, we therefore first evaluate its performance for connections with delays that equal the simulation time step. We compare the results to networks with ordinary STDP synapses, again setting all learning rates to zero to maintain the same network state across different types of plasticity. Naturally, the processing of the membrane potential information makes the Urbanczik-Senn plasticity less efficient to simulate than networks with ordinary STDP synapses (Figure 11). Note that the absolute numbers of simulation times are not directly comparable to simulations with Clopath plasticity (Figure 9) as network sizes are smaller here (Supplementary Table 5). Networks with small and large in-degrees behave qualitatively similar: given the long continuous history that needs to be stored and read out, the event-driven scheme does not significantly outperform the time-driven scheme (Figures 11A,B). In the network with small in-degree, the time-driven scheme is even slightly faster (Figure 11A). This behavior reverses for large in-degrees as the number of synapse calls grows stronger than the length of the history (Figure 11B). However, given that the length of the history is so critical in this rule, the compression algorithm can in both cases achieve a significant increase in performance (Figures 11A,B). This performance increase is larger the smaller the in-degree, as the compressed history becomes shorter (Figure 11A). Due to current NEST specifics (see section 3.2.2), the compression algorithm cannot be used in settings with delays that are larger than the simulation time step (Figure 11C): Here, as expected, the time-driven scheme becomes faster than in the *d* = *h* case, but it is in general still comparable in performance to the event-driven scheme. The latter is therefore the method of choice for simulations with delayed connections; for zero-delay connections, the compression algorithm performs best. Whether the history readout is done via linear iteration or via computing positions of history entries has no significant impact on the performance (Figure 11D). Therefore, the simple linear iteration is integrated in NEST 2.20.1.

**Figure 12:**
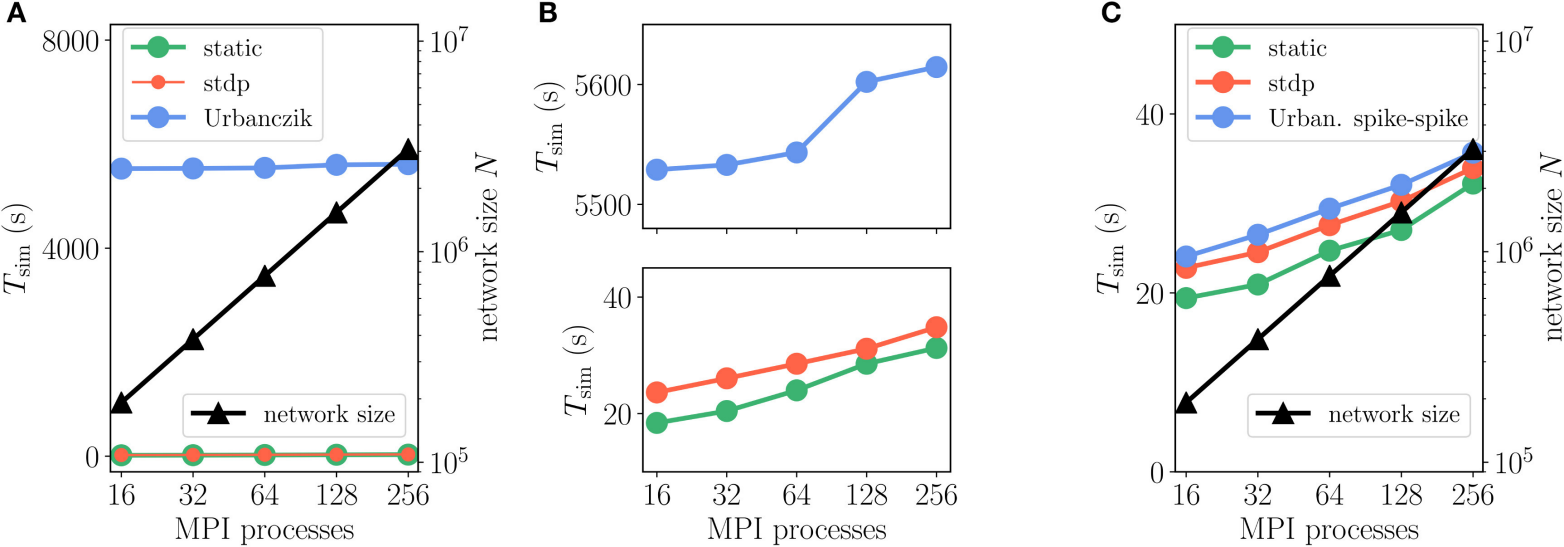
Scaling of simulation times *T*_sim_ with network size for 2 *s* of biological time: Urbanczik-Senn plasticity. Same weak scaling as in Figure 10. **(A)** Event-driven Urbanczik-Senn rule (blue) compared to static (green) and STDP synapse (red). On the scale of the vertical axis the red curve (STDP synapses) falls on top of the green curve (static synapses), indicated by finer line width and marker size of the former. **(B)** Same simulation time data as in **(A)** but with a smaller range on the vertical axis. Upper panel: enlargement of Urbanczik-Senn data. Lower panel: enlargement of data for static and STDP synapses. **(C)** Spike-spike version of the Urbanczik-Senn rule compared to static and STDP synapse. Network and simulation parameters as in Supplementary Table 5 with in-degree *K* = 5, 000. For all simulations each compute node runs one MPI process with 24 threads.

We furthermore employ a weak-scaling setup with excitatory-inhibitory networks of increasing size and fixed in-degree *K* = 5, 000 (Figures 12A,B, and Supplementary Table 5). Apart from a constant offset, the scaling of simulation time *T*_sim_ for updating neurons and synapses is similar for Urbanczik, static and STDP synapses. With increasing network size *N* and proportionally increasing number of MPI processes, *T*_sim_ rises only slightly (Figure 12B), indicating almost ideal weak-scaling behavior. The constant offset in *T*_sim_ is larger than for Clopath synapses as the Urbanczik-Senn rule requires longer histories of membrane potentials and a more extensive history management.

### 3.5. Conclusions

The analyses of the Clopath and the Urbanczik-Senn plasticity as prototypical examples for rules that rely on storage of discontinuous vs. continuous histories show that the former are much faster to simulate, in particular for large networks that require distributed computing. For discontinuous histories, the event-driven scheme is most generally applicable and efficient, which makes corresponding rules easy to integrate into modern simulators with event-based synapses. The performance gap between the different rules should be kept in mind in the design of new learning rules. Furthermore, it is worthwhile to test modifications of existing learning rules to decrease the amount of stored information.

For illustration, we here test a spike-based alternative to the original Urbanczik-Senn rule, where we replace the rate prediction ϕ (*V*_*i*_ (*t*)) in V^*^ of (15) by a noisy estimate, which we generate by a non-homogeneous Poisson generator with rate ϕ (*V*_*i*_(*t*)), see Appendix Section 5.7. The prediction error then results in a comparison of somatic and dendritic spikes, *s*_*i*_ and $s_{i}^{\text{dend}}$, respectively; it is therefore purely based on point processes. In terms of storage and computations, the rule thereby becomes similar to ordinary STDP [cf. (5)]. This becomes apparent in the weak-scaling experiment in Figure 12C, which shows that the modification of the learning rule results in a speedup of a factor 10 to 30 arriving essentially at the same run time as the ordinary STDP rule.

**Figure 13:**
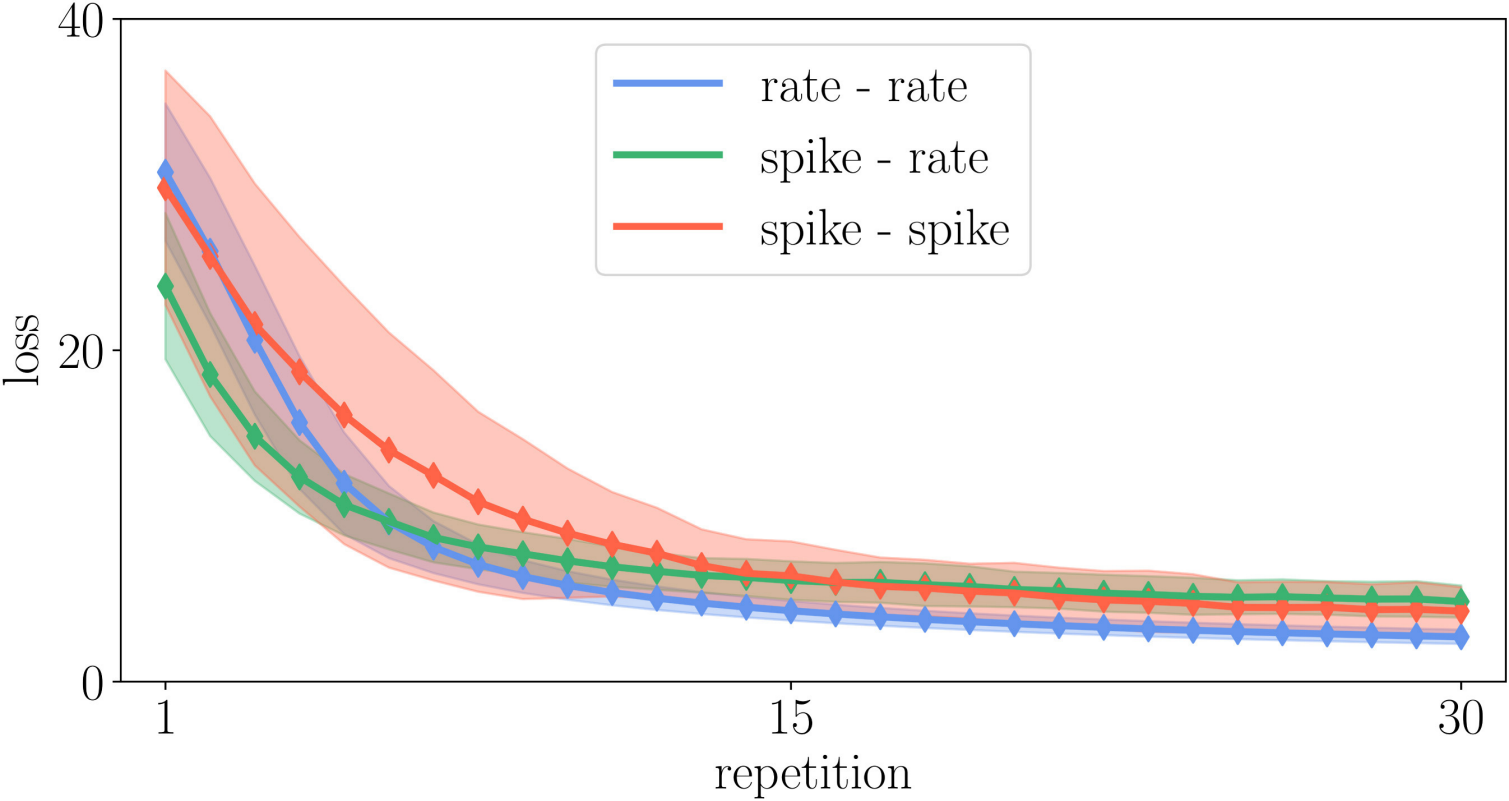
Comparison of learning curves in the experiment described in Appendix Section 5.6 for different variants of the Urbanczik-Senn plasticity rule. The loss is averaged over 128 trials of different input patterns. Solid curves denote the mean value and the shaded area the corresponding standard deviation of the loss.

When changing learning rules to improve the efficiency of an implementation, the question is in how far the modified rule, in our example including the noisy estimate of the dendritic prediction, still fulfills the functionality that the original rule was designed for. Generally, without control of the error any simulation can be made arbitrarily fast. Therefore, Morrison et al. (2007b) define efficiency as the wall-clock time required to achieve a given accuracy. We test in Figure 13 whether the dynamics is still robust enough to achieve proper learning and function in the reproduced task of Figure 8. The learning works as well as in the original Urbanczik-Senn rule. However, given the simplicity of the chosen task, this result may not generalize to other more natural tasks. We leave a more detailed investigation of this issue to future studies. The basic exploration here, however, illustrates how taking into account the efficiency of implementations can guide future development of learning rules to make them practically usable for large-scale simulations of brain networks.

## 4. Discussion

This work presents efficient algorithms to implement voltage-based plasticity in modern neural network simulators that rely on event-based updates of synapses (for a review, see Brette et al., 2007). This update scheme restricts function calls of synapse code to time points of spike events and thereby improves performance in simulations of biologically plausible networks, where spike events at individual synapses are rare and the total number of synapses is large compared to the number of neurons. While our framework has no restrictions on the postsynaptic voltage-dependence of the learning rule, a particular focus of this work is on those plasticity rules, where synapses rely on an extended history of membrane potentials and therefore continuous information of state variables of postsynaptic cells to update their strength. This dependence naturally suggests a time-driven update of synapses. Instead, we here propose an efficient archiving of voltage traces to enable event-based synapse updates and detail two schemes for storage, read out and post-processing of time-continuous or discontinuous information. We show their superior performance with respect to time-driven update both theoretically and with a reference implementation in the neural network simulation code NEST for the rules proposed in Clopath et al. (2010) and Urbanczik and Senn (2014).

Event-driven update schemes for voltage-based plasticity come at the expense of storing possibly long histories of a priori continuous state variables. Such histories not only require space in memory but they also affect the runtime of simulations, which we focus on here. The time spent for searching and post-processing the history to calculate weight updates increases with increasing length, and these operations have to be done for each synapse. Therefore, in addition to an ordinary event-driven scheme, we devised a compression scheme that becomes superior for long histories as occurring in the Urbanczik-Senn rule. In particular for networks with small in-degrees or synchronous spiking, the compression scheme results in a shorter history. It further reduces the total amount of computations for weight changes by partially re-using results from other synapses thereby avoiding multiple processing of the history. For short histories as occurring in the Clopath rule, the compression results in unnecessary overhead and an increase in history size as one entry per last presynaptic spike time needs to be stored instead of a discontinuous membrane potential around sparse postsynaptic spike events. We here, for simplicity, contrasted time- and event-driven update schemes. However, further work could also investigate hybrid schemes, where synapses are not only updated at spike events, but also on a predefined and coarse time grid to avoid long histories and corresponding extensive management. A similar mechanism is used in Kunkel et al. (2011) to implement a normalization of synaptic weights. The corresponding technical details can be found in Kunkel (2015, ch. 5.2).

The storage and management of the history as well as complex weight change computations naturally reduce the performance of simulations with voltage-based plasticity in comparison to static or STDP synapses. The latter only require information on spike times which is much less data compared to continuous signals. Nevertheless, given that the Clopath rule is based on thresholded membrane potentials and consequently short, discontinuous histories, the performance and scaling of the event-driven algorithms is only slightly worse than for ordinary STDP. Time-driven implementations cannot employ this model feature and update weights also in time steps where no adjustment would be required, leading to significantly slower simulations. The performance gain of using event-driven schemes is less pronounced for the Urbanczik-Senn rule as, by design, histories are typically long. In this case, the compression scheme naturally yields better results in terms of runtime. Our own modification of the Urbanczik-Senn rule only requires storage of sparsely sampled membrane potentials, giving rise to the same performance as STDP. Generally, an algorithm is faster if it requires fewer computations. However, opportunities for vectorization and cache efficient processing, outside of the scope of the present manuscript, may change the picture.

We here chose the Clopath and the Urbanczik-Senn rule as two prototypical models of voltage-based plasticity. While both rules describe a voltage dependence of weight updates, their original motivation as well as their specific form are different: The Clopath rule refines standard STDP models to capture biologically observed phenomena such as frequency dependence of weight changes (Sjöström et al., 2001). For this it is sufficient to take into account membrane potential traces in the vicinity of spike events, leading to storage of time-discontinuous histories in our implementation. In contrast, the Urbanczik-Senn rule is functionally inspired by segregating dendritic and somatic compartments of cells and using the difference between somatic output and dendritic prediction as a teacher signal for dendritic synapses. The teacher signal is by construction never vanishing, imposing the need to store a time-continuous history. The original publications of both rules had a great and long-lasting impact on the field. The Clopath rule has been used in a variety of studies (Clopath and Gerstner, 2010; Ko et al., 2013; Litwin-Kumar and Doiron, 2014; Sadeh et al., 2015; Bono and Clopath, 2017; Maes et al., 2020), partly in modified versions which are, however, still compatible with the here presented simulation algorithms. The same holds for the Urbanczik-Senn rule (Brea et al., 2016; Sacramento et al., 2018).

The current implementation, which is published and freely available in NEST 2.20.1, supports an adaptive exponential integrate-and-fire and a Hodgkin-Huxley neuron model for the Clopath rule. The former is used in the original publication (Clopath et al., 2010) and the latter appears on ModelDB (Hines et al., 2004) in code for the Clopath rule for the NEURON simulator (Hines and Carnevale, 2001). For the Urbanczik-Senn rule, NEST currently supports the two-compartment Poisson model neuron of the original publication (Urbanczik and Senn, 2014). A three-compartment version as used in Sacramento et al. (2018) or other models are straight forward to integrate into the current simulation framework. However, with voltage-based plasticity rules, borders between neurons and synapses become blurred as these rules often depend on specifics of the employed neuron models rather than only spike times as for standard STDP. Consequently, archiving nodes might need to have specific functionalities, which, in light of the zoo of existing neuron models, could easily lead to a combinatorial explosion of code. These problems can in future be overcome with automatic code generation using NESTML that only creates and compiles code that is needed for the specified model simulations (Plotnikov et al., 2016).

While the here presented implementation refers to the neural network simulator NEST (Gewaltig and Diesmann, 2007), the proposed algorithms and simulation infrastructure are compatible with any network simulator with event-driven update of synapses, such as for example, NEURON (Lytton et al., 2016, cf. ch. 2.4) and Brian2 (Stimberg et al., 2014). Furthermore, applicability is not restricted to the Clopath and Urbanczik-Senn rule, but the framework can be adapted to any other learning rule that relies on state variables of postsynaptic neurons. State variables hereby not only encompass membrane potentials such as for example, in the LCP rule by Mayr and Partzsch (2010), the Convallis rule by Yger and Harris (2013), the voltage-triple rule by Brea et al. (2013), the MPDP rule by Albers et al. (2016), the voltage-gated learning rules by Brader et al. (2007), Sheik et al. (2016), Qiao et al. (2015), Diederich et al. (2018), and Cartiglia et al. (2020), or the branch-specific rule by Legenstein and Maass (2011), but also, for example, firing rates of stochastic neuron models or rate models (Brea et al., 2016; Sacramento et al., 2018), or other learning signals (Neftci et al., 2017; Bellec et al., 2020). The infrastructure in NEST allows for the storage of time-continuous and discontinuous histories and therefore poses no restrictions on the dependence of the learning rule on the postsynaptic state variables. The here developed machinery could be also used to store external teacher signals that are provided to model neurons by stimulation devices mimicking brain or environmental components not explicitly part of the model. Since synapses are located at the compute process of the postsynaptic neuron, readout of state variables from presynaptic neurons comes with large costs in simulations on distributed computing architectures and is therefore not considered here. Due to specifics of the present NEST code in spike delivery, the event-driven compression proposed here is only applicable in NEST for delays that equal the simulation time step. Such a restriction can be readily overcome in a simulation algorithm that performs a chronological update of synapses.

In general, one has to distinguish two types of efficiency in the context of simulating plastic networks: Firstly, the biological time it takes the network to learn a task by adapting the weights of connections. Secondly, the wall-clock time it takes to simulate this learning process. Both times crucially depend on the employed plasticity rule. In this study, we focus on the wall-clock time and argue that this can be optimized by designing learning rules that require storing only minimal information on postsynaptic state variables. Ideally, the plasticity rule contains unfiltered presynaptic or postsynaptic spike trains to reach the same performance as in ordinary STDP simulations. This amounts to synapses requiring postsynaptic state variables only at the time of spike events. The Clopath and Urbanczik-Senn rule capture the dependence of synaptic weights on the postsynaptic membrane potential in a phenomenological manner. The dependence on the voltage history observed in biological synapses (Artola et al., 1990; Ngezahayo et al., 2000) is an indirect effect mediated by receptors and channels with voltage-dependent dynamics (see Clopath et al., 2010, and references therein). Modeling these complex dynamics explicitly and evolving corresponding state variables in the postsynaptic neurons would enable a readout of these quantities only at spike times and thereby remove the need to maintain explicit histories. If phenomenological rules, however, need to capture the pre- and post-spike dynamics of postsynaptic membrane potentials explicitly, it is beneficial to make use of thresholds as in the example of the Clopath rule as these yield short, time-discontinuous histories. Reducing the amount of information available for synapses to adjust their weights can in general slow down the learning. We present a modification of the Urbanczik-Senn rule where the dendritic prediction of the somatic firing contains an additional sampling step with Poisson spike generation. This modification significantly reduces the simulation time. For the here presented simple task, learning speed is largely unaffected, but generally a performance decrease is to be expected when error signals become more noisy. Therefore, there is a trade-off between learning speed and simulation speed, which should be considered in the design process of new learning rules. Complex voltage-based plasticity rules have been simplified and turned into voltage-gated learning rules to make them compatible with event-based synapse updates: Cartiglia et al. (2020) propose a modification of the Urbanczik-Senn rule underlying the model in Sacramento et al. (2018). This simplification only requires postsynaptic membrane potentials at the time of spike events, which makes the rule much more efficient to simulate and applicable to neuromorphic hardware. Bono and Clopath (2017) simplify the Clopath rule in an analogous fashion to allow for its event-based simulation in the spiking network simulator Brian2, see documentation at https://brian2.readthedocs.io/en/stable/examples/frompapers.Clopath_et_al_2010_homeostasis.html (Stimberg et al., 2014). Our general framework supports systematic testing of such simplifications in terms of simulation performance and functionality.

For the plasticity rules by Clopath et al. (2010) and Urbanczik and Senn (2014), we present a highly scalable reference implementation that is published and freely available in NEST 2.20.1. The parallelism of the NEST implementation enables simulations of plastic networks of realistic size on biologically plausible time scales for learning. The field of computational neuroscience recently entered a new era with the development of large-scale network models (Markram et al., 2015; Schmidt et al., 2018; Billeh et al., 2020). Emulating the dynamics of cortical networks, such models are so far restricted to static connections. We here provide simulation algorithms for plasticity mechanisms that are required for augmenting such complex models with functionality. It is our hope that incorporating both biologically and functionally inspired plasticity models in a single simulation engine fosters the exchange of ideas between communities toward the common goal of understanding system-level learning in the brain.

## Data Availability Statement

The datasets presented in this study can be found in online repositories. The names of the repository/repositories and accession number(s) can be found at: Stapmanns et al. (2021).

## Code Availability

The reference implementation for the event-driven update scheme of synapses with Clopath and Urbanczik-Senn plasticity was reviewed by the NEST initiative and is publicly available in NEST 2.20.1. The PyNEST code for model simulations and Python scripts for the analysis and results are fully available at: Stapmanns et al. (2021).

## Author Contributions

JS and JH wrote the simulation code, the plotting scripts, and performed the NEST simulations for the HPC performance measurements. JS and DD worked out the details of the theoretical analysis of the different algorithms. JS was supervised by MH, MD, and DD. JH was supervised by MB. All authors jointly did the conceptual work and wrote the paper.

## Conflict of Interest

The authors declare that the research was conducted in the absence of any commercial or financial relationships that could be construed as a potential conflict of interest.

## 5. Appendix

### 5.1. Analytical Integration in Urbanczik-Senn Rule

To derive (17) it is convenient to first investigate Δ*W*_*ij*_(0, *t*) and Δ*W*_*ij*_(0, *T*) and then compute Δ*W*_*ij*_(*t, T*) = Δ*W*_*ij*_(0, *T*) − Δ*W*_*ij*_(0, *t*). Assuming that the simulation starts at *t* = 0, the weight change from the start to time *t* is given by

$$\begin{array}{l} \Delta W_{ij}\left(0,t\right)=\eta \int_{0}^{t}dt^{\prime }\int_{0}^{t^{\prime }}dt^{\prime \prime }\;\kappa \;\left(t^{\prime }-t^{\prime \prime }\right)\;V_{i}^{*}\;\left(t^{\prime \prime }\right)\;s_{j}^{*}\;\left(t^{\prime \prime }\right)\\ \;=\eta \int_{0}^{t}dt^{\prime \prime }\int_{t^{\prime \prime }}^{t}dt^{\prime }\;\kappa \;\left(t^{\prime }-t^{\prime \prime }\right)\;V_{i}^{*}\;\left(t^{\prime \prime }\right)\;s_{j}^{*}\;\left(t^{\prime \prime }\right)\\ \;=\eta \int_{0}^{t}dt^{\prime \prime }\;\left[\;\tilde{\kappa }\;\left(t-t^{\prime \prime }\right)-\tilde{\;\kappa }\;(0)\right]\;V_{i}^{*}\;\left(t^{\prime \prime }\right)\;s_{j}^{*}\;\left(t^{\prime \prime }\right)\\ \;=\eta \;[-I_{2}\left(0,t\right)+I_{1}\left(0,t\right)] \end{array}$$

where we exchanged the order of integration from the first to the second line. In the third line we introduced $\tilde{\kappa }\;\left(t\right)$ defined by $\kappa \;(t)=\frac{\partial }{\partial t}\tilde{\kappa }\;(t)$ and in the fourth line we defined the two integrals

$$\begin{array}{l} I_{1}\left(t_{1},t_{2}\right)=-\int_{t_{1}}^{t_{2}}dt^{\prime }\;\tilde{\kappa }\;\left(0\right)\;V_{i}^{*}\;\left(t^{\prime }\right)\;s_{j}^{*}\;\left(t^{\prime }\right),\\ I_{2}\left(t_{1},t_{2}\right)=-\int_{t_{1}}^{t_{2}}dt^{\prime }\;\tilde{\kappa }\;\left(t-t^{\prime }\right)\;V_{i}^{*}\;\left(t^{\prime }\right)\;s_{j}^{*}\;\left(t^{\prime }\right). \end{array}$$

In case of the Urbanczik-Senn rule

$$\;\tilde{\kappa }\;\left(t\right)=-e^{-\frac{t}{\tau_{\;\kappa \;}}}$$

which implies the identities

$$\begin{array}{l} I_{1}\;(t_{1},t_{2}+\Delta t)=I_{1}\;(t_{1},t_{2})+I_{1}\;(t_{2},t_{2}+\Delta t),\\ I_{2}\;(t_{1},t_{2}+\Delta t)=e^{-\frac{t_{2}-t_{1}}{\tau_{\;\kappa \;}}}I_{2}\;(t_{1},t_{2})+I_{2}\;(t_{2},t_{2}+\Delta t), \end{array}$$

which we use to write the weight change from *t* to *T* as

$$\begin{array}{l} \Delta W_{ij}\left(t,T\right)=\Delta W_{ij}\left(0,T\right)-\Delta W_{ij}\left(0,t\right)\\ \;=\eta \;\left[-I_{2}\;(0,T)+I_{1}\;(0,T)+I_{2}\;(0,t)-I_{1}\;(0,t)\right]\\ \;=\eta \;\left[I_{1}\;(t,T)-I_{2}\;(t,T)+I_{2}\;(0,t)\left(1-e^{-\frac{T-t}{\tau_{\;\kappa \;}}}\right)\right]. \end{array}$$

This is the the result (17).

### 5.2. Voltage Clamping of the Adaptive Exponential Integrate-and-Fire Model

For the Clopath rule the change of the synaptic weight strongly depends on the excursion of the membrane potential *V*_*m*_ around a spike of the postsynaptic neuron which causes ū_±_ to cross the respective thresholds θ_±_ so that (12) and (13) yield non-vanishing results. Within the original neuron model Brette and Gerstner (2005) *u* is reset immediately after it reached the spiking threshold so that the shape of the action potential is not described accurately. In our NEST implementation of aeif_psc_delta_clopath we adapted the approach of the reference implementation on ModelDB (Hines et al., 2004) and introduced a clamping of *u* to a fixed value *V*_clamp_ for a period of *t*_clamp_ before it is reset. The reference implementation is restricted to a simulation resolution of exactly 1 ms and sets *u* to two different values for the two subsequent simulation steps after a spike. To be independent of the resolution of the simulation, the implementation in NEST uses a constant *V*_clamp_. In the simulations we set *t*_clamp_ to 2 ms and *V*_clamp_ to 33 mV. These values are chosen to match the behavior of the reference implementation.

### 5.3. History Management

There are three points that need to be considered in the context of history management: First, which information needs to be stored. Second, how to search and read out the history. Third, how to identify and remove information that is no longer needed. The first and third point mainly affect memory usage, while the second point impacts the simulation time as searching within shorter histories is faster.

There are four different histories to which our considerations apply. The one to store the membrane potential $V_{i}^{*}$, the compressed history Δ*W*_*i*_(*t*_*LS*_, *T*) used only for the compressed event-driven scheme, the history to store the spike times *s*_*i*_ of the postsynaptic neuron (also used for ordinary STDP), and finally one might need a history that stores the last spike time for every incoming synapse (see below for details).

#### 5.3.0.1. Adding Information to the History

This paragraph concerns only the history that stores the time trace of $V_{i}^{*}$. In every time step of the simulation, neurons call the protected function write_history() of the archiving node and pass the current value of the (low-pass filtered) membrane potential. The archiving node then computes the derived quantities $V_{i}^{*}$ or combinations of $V_{i}^{*}$ and $s_{i}^{*}$, and saves them in the history, which is of type vector. It is more efficient to do the computations inside the archiving node and not in the synapse for two reasons: Firstly, the computation is done only once and then used for all incoming synapses. This way no direct exchange of information between different synapses is required. Secondly, the archiving node does not need to store the raw membrane potentials before readout, but can directly store the derived quantities $V_{i}^{*}$, which reduces the memory consumption, especially in cases where only a non-continuous history is needed.

#### 5.3.0.2. Readout of Information From the History

Let's assume *t*_*LS*_ and *t*_*S*_ be the times of the last and the current spike of a synapse. At time *t*_*S*_ that synapse then needs to request a part from *t*_1_ = *t*_*LS*_ − *d* to *t*_2_ = *t*_*S*_ − *d* > *t*_1_ of the history that ranges from *t*_*start*_ < *t*_1_ to *t*_*end*_ > *t*_2_. This part is shifted with respect to the spike times by a delay *d* which models the time of signal propagation from the postsynaptic soma back to the synapse. The software framework NEST of our reference implementation uses only one variable to represent the delay from synapse to soma and the delay in the opposite direction. Consequently, when a spike arrives at the synapse of the postsynaptic neuron, the synapse sees a membrane potential from the past. In case every time step of the simulation adds a new entry to the history, one can easily compute the positions of the entries corresponding to *t*_1/2_ by just knowing *t*_*start*_ and *t*_*end*_. As pointed out in section 3.2 this is the case for the Urbanczik-Senn plasticity rule. If the history is not continuous in time, like in case of the Clopath rule, this scheme is not applicable. Instead, we add a time stamp *s* as an additional variable to each entry and search for those with the smallest/greatest *s* within the interval (*t*_1_, *t*_2_) using e.g., a linear or a binary search. Searching for the positions that define the start and the end of the requested interval is slower than computing them directly. Nevertheless, a non-continuous history can lead to a large acceleration of simulations as we discussed in case of the Clopath rule (section 3.4.1). Here, only values of the membrane potential in the vicinity of a spike of the postsynaptic neuron are needed so that neglecting the majority of values in between leads to a non-continuous history but saves memory.

Technically, the archiving node contains a function called get_history() which expects two iterators start and finish and two times *t*_1_ and *t*_2_. When executed, the function sets the iterators to point to the correct entries of the history of the postsynaptic neuron corresponding to *t*_1_ and *t*_2_, respectively. Having received the correct position of the pointers, the synapse evaluates the integral (6). In the event-driven compression scheme, the integration (11) is not done inside the synapse but inside the archiving_node. The reason for this is that the compressed history Δ*W*_*i*_(*t*_*LS*_, *t*_*S*_), which is updated in case of an incoming spike, is stored inside the archiving_node. This way no exchange of information is needed. The synapse only triggers the updating process by calling the function compress_history() of the archiving_node. Internally, the archiving_node can use get_history() to obtain the part of the history that has to be integrated. Even though the linear search a priori might seem less efficient than a binary search or direct computation of the position, it turns out that it has an advantage in that it iterates consecutively over the history entries which can be employed to identify data no longer needed. Therefore, especially for short histories a simple iteration that comes without any overhead is fastest (see section 3.4.1).

#### 5.3.0.3. Removing Information From the History

To prevent the history from occupying an unnecessary amount of memory, it is crucial to have a mechanism to delete those entries that have been used by all incoming synapses. The simplest implementation to identify these entries is to add one additional variable to each entry called *access counter* initialized to zero when the entry is created. When a synapse requests a part from *t*_1_ to *t*_2_ of the history, the algorithm iterates over all entries *t*_1_ < *t* < *t*_2_ and increases the access counters by one. After the update of the synaptic weight all entries whose access counters are equal to the number of incoming synapses are deleted. This scheme can be combined easily with a linear search starting the iteration from the oldest entry of the history.

For long histories a linear search is inefficient and should be replaced by a binary search or direct computation of positions if applicable. To avoid iteration within long histories, we replace access counters by a vector that stores the last spike time *t*_*LS*_ for every incoming synapse. If a synapse delivers a spike, it updates its entry in that vector by replacing *t*_*LS*_ by the time stamp of the current spike. After each weight update, searching the vector for the smallest *t*_*LS*_ allows us to safely remove all membrane potentials with time stamps *t* < min({*t*_*LS*,*i*_}). In practice, we can further improve this mechanism with two technical details. Firstly, *n* incoming spikes with the same time stamp can share the same entry *t*_*LS*_ which we then have to provide with a counter that goes down from *n* to zero in steps of one whenever one of the *n* synapses sends a new spike for a time *t* > *t*_*LS*_. Secondly, we can avoid the search for the smallest *t*_*LS*_ by making sure that the entries *t*_*LS*_ are in chronological order. This can be easily achieved if a synapse does not update its entry in the vector but removes it and appends a new one at the end of the vector.

### 5.4. Implementation of Experiments Using Clopath Rule

#### 5.4.1. Spike Pairing Experiment

The setup of the spike pairing experiment from Clopath et al. (2010) presented in Figures 7A,B consists of two neurons connected via a plastic synapse. The pre- and postsynaptic neuron are forced to spike with a time delay of Δ*t* by stimulation with spike_generators at times $t_{\text{pre}}^{\left(i\right)}=t^{\left(i\right)}$ and $t_{\text{post}}^{\left(i\right)}=t^{\left(i\right)}+\Delta t$, respectively. A positive time shift Δ*t* > 0 refers to the presynaptic neuron spike before the postsynaptic one (pre-post pairing, solid lines in Figure 7) and vice versa. Spike pairs $\left(t_{\text{pre}}^{\left(i\right)},t_{\text{post}}^{\left(i\right)}\right)$ are induced with frequency $f_{pair}=\frac{1}{t^{\left(i+1\right)}-t^{\left(i\right)}}$ and the weight change of the synapse is measured after a set of five pairs. In our simulation using NEST the presynaptic neuron is modeled as a parrot_neuron and the postsynaptic neuron is either of type aeif_psc_delta_clopath or hh_psc_alpha_clopath. In NEST parrot_neurons are model neurons that emit a spike whenever they receive one. In this setup they are required because devices like spike_generators support only static synapses in NEST so that we cannot connect the postsynaptic neuron directly to the spike_generator via a plastic synapse. The initial weight of the clopath_synapse connecting the two neurons is given by *w*_*init*_. In this experiment we use the Clopath rule with fixed amplitude *A*_*LTD*_. A list with all the parameters can be found in Supplementary Table 1.

#### 5.4.2. Emergence of Strong Bidirectional Couplings

In this experiment after Clopath et al. (2010), a small network of *N*_*I*_ inhibitory and *N*_*E*_ excitatory neurons subject to an external input develops strong bidirectional couplings between neurons of the excitatory population. The input is given by *N*_*p*_ Poisson spike trains with rates

(18) $$\begin{array}{l} f_{p}^{\left(j\right)}=A_{p}e^{-\frac{{\left(j-\mu_{p}\right)}^{2}}{2\sigma_{p}^{2}}}+c_{p}, \end{array}$$

where *j* = 1, …, *N*_*p*_. The center μ_*p*_ of the Gaussian is drawn randomly from a set *s*_*p*_ of possible values and a new value is drawn after each time interval *t*_μ_. The total number of intervals is *N*_μ_. In our simulation with NEST we used aeif_psc_delta model neurons with the same parameters (cf. Supplementary Table 3) for both the inhibitory and the excitatory population. The simulation is divided into *N*_μ_ intervals between which the rates of the *N*_*p*_ poisson_generators are set according to (1). The poisson_generators are connected in a one-to-one manner to *N*_*p*_ parrot_neurons which in turn are connected to the network. The details of the latter connectivity can be found in Supplementary Table 2. In NEST a poisson_generator that is connected to several target model neurons generates an independent Poisson spike train for each of these neurons. Thus, the intermediate step via parrot_neurons is required to provide neurons in the network with common Poisson inputs. Moreover, a direct connection from a device like a poisson_generator to a model neuron via a plastic synapse is not possible in NEST. In this experiment, the Clopath rule with homeostasis (time dependent prefactor for LTD, cf. section 5.5) is used. Figure 7C shows the weights of the synapses among the excitatory population after the simulation.

### 5.5. Implementation of Homeostasis $A_{\text{LTD}}\left(\overset{-}{ū}\right)$

For the network simulations presented in Clopath et al. (2010), the authors use a sightly modified version of the Clopath rule defined in (12): The constant factor *A*_*LTD*_ is replaced by a voltage dependent term

$$A_{LTD}\;(\overset{-}{ū})=A_{LTD}{\;(\frac{\overset{-}{ū}}{u_{ref}})}^{2}$$

to take into account homeostatic processes. The quantity $\overset{-}{ū}$ is a temporal average of the quantity ū _−_(*t*) over a time window of *T* = 1 *s* and *u*_*ref*_ is a reference value. An exact temporal average requires storing the time trace of ū_−_ (*t*) for the entire interval *T*. This would cancel the advantage of keeping only a sparse history as discussed in 3.2.3.2 where storage of time traces is needed only in the vicinity of spikes. Therefore, deviating from the original work by Clopath et al. (2010), we implement an additional low-pass filtering $\overset{-}{ū}\left(t\right)=\left(\kappa_{\text{low}}*{ū}_{-}\right)\left(t\right)$ with an exponential kernel $\kappa_{\text{low}}\left(t\right)=H\left(t\right)\exp\left(-t/\bar{\bar{\tau }}\right)$ instead. Like ū_±_, $\overset{-}{ū}$ is passed as an additional state variable to the solver.

### 5.6. Implementation of Experiment Using Urbanczik-Senn Rule

In the simulation experiment shown in Figure 8 the dendrite of a conductance-based two-compartment model neuron receives a spike pattern of duration *T* as an input via plastic synapses. The pattern consists of *N*_*p*_ independent Poisson spike trains with a firing rate *f*_*p*_. For learning, the pattern is repeated *N*_*rep*_ times. Dendritic synapses adapt their weights so that after learning the somatic membrane potential *U* and the dendritic prediction $V_{w}^{*}$ follow a matching potential *U*_*M*_. The latter is created by somatic input via two spike_generators that are connected via a static excitatory or inhibitory connection, respectively. Both spike generators send spikes in every simulation step. Inhibitory input spikes have a constant weight to generate a constant somatic inhibitory conductance *g*_*I*_. Excitatory spikes have a modulated weight to generate a periodic excitatory conductance *g*_*E*_. The input to the dendritic compartment is provided by *N*_*p*_ spike_generators each of which is connected to one parrot_neuron which in turn is connected to the dendrite via a plastic urbanczik_synapse. The intermediate parrot_neurons are required since in NEST the spike_generators can have only static synapses as outgoing connections. The spike times of the spike_generators are set to repeatedly generate the spike pattern created before the start of the actual simulation. The neuron's state variables are read out by a multimeter and the synaptic weights by a weight_recorder.

### 5.7. Experiment With Modified Version of the Urbanczik-Senn Rule

The weight change of the Urbanczik-Senn rule as presented in section 2.5 in line with the original publication is driven by the prediction error

$$V_{i}^{*}\;=\left(s_{i}-\phi \left(V_{i}\right)\right)\;h\;\left(V_{i}\right),$$

where *s*_*i*_ is the somatic spike train and *V*_*i*_ the dendritic prediction of the somatic membrane potential *U*_*i*_. Instead of integrating over the difference between the spike train and the rate ϕ (*V*_*i*_) (spike-rate), one can derive two variants

$$\begin{array}{l} \;V_{i}^{*}\;=\left(s_{i}-s_{i}^{dend}\right)\;h\;\left(V_{i}\right)\;\left(spike-spike\right)\;and\\ \;V_{i}^{*}\;=\left(\phi \;\left(U_{i}\right)-\phi \;\left(V_{i}\right)\right)\;h\;\left(V_{i}\right)\;\left(rate-rate\right). \end{array}$$

In the first one (spike-spike) we replaced the dendritic rate prediction by a noisy realization $s_{i}^{\text{dend}}$ using an inhomogeneous Poisson process with rate ϕ (*V*_*i*_). In the second one (rate-rate) the somatic spike train is replaced by the rate of the underlying Poisson process which is computed by applying the rate function ϕ to the somatic potential *U*_*i*_. The learning of a matching potential *U*_*M*_ as described in section 3.3 also works in these two cases. Figure 13 shows the learning curve for all three variants of the Urbanczik-Senn rule. The loss is defined as the average mismatch between *U*_*i*_ and *U*_*M*_ averaged over one period *T*_*p*_ of the input pattern

$$\frac{1}{T_{p}}\int dt\;{\left(U\;\left(t\right)-U_{M}\;(t)\right)}^{2}.$$

The decrease of the loss as a function of the pattern repetitions has a similar shape for all three variants with a significantly higher variance in case of the spike-spike version.

## References

[B1] Albers C. Westkott M. Pawelzik K. (2016). Learning of precise spike times with homeostatic membrane potential dependent synaptic plasticity. PLoS ONE 11:e0148948. 10.1371/journal.pone.0148948 26900845 PMC4763343

[B2] Artola A. Bröcher S. Singer W. (1990). Different voltage dependent thresholds for inducing long-term depression and long-term potentiation in slices of rat visual cortex. Nature 347, 69–72. 10.1038/347069a0 1975639

[B3] Bellec G. Scherr F. Subramoney A. Hajek E. Salaj D. Legenstein R.. (2020). A solution to the learning dilemma for recurrent networks of spiking neurons. Nat. Commun. 11:3625. 10.1038/s41467-020-17236-y 32681001 PMC7367848

[B4] Bi G. Poo M. (1998). Synaptic modifications in cultured hippocampal neurons: Dependence on spike timing, synaptic strength, and postsynaptic cell type. J. Neurosci. 18, 10464–10472. 10.1523/JNEUROSCI.18-24-10464.1998 9852584 PMC6793365

[B5] Billeh Y. N. Cai B. Gratiy S. L. Dai K. Iyer R. Gouwens N. W.. (2020). Systematic integration of structural and functional data into multi-scale models of mouse primary visual cortex. Neuron 106, 388–403.e18. 10.1016/j.neuron.2020.01.040 32142648

[B6] Bono J. Clopath C. (2017). Modeling somatic and dendritic spike mediated plasticity at the single neuron and network level. Nat. Commun. 8, 1–17. 10.1038/s41467-017-00740-z 28951585 PMC5615054

[B7] Brader J. M. Senn W. Fusi S. (2007). Learning real world stimuli in a neural network with spike-driven synaptic dynamics. Neural Comput. 19, 2881–2912. 10.1162/neco.2007.19.11.2881 17883345

[B8] Brea J. Gaal A. T. Urbanczik R. Senn W. (2016). Prospective coding by spiking neurons. PLoS Comput. Biol. 12:e1005003. 10.1371/journal.pcbi.1005003 27341100 PMC4920376

[B9] Brea J. Senn W. Pfister J.-P. (2013). Matching recall and storage in sequence learning with spiking neural networks. J. Neurosci. 33, 9565–9575. 10.1523/JNEUROSCI.4098-12.2013 23739954 PMC6619697

[B10] Brette R. Gerstner W. (2005). Adaptive exponential integrate-and-fire model as an effective description of neuronal activity. J. Neurophysiol. 94, 3637–3642. 10.1152/jn.00686.2005 16014787

[B11] Brette R. Rudolph M. Carnevale T. Hines M. Beeman D. Bower J. M.. (2007). Simulation of networks of spiking neurons: a review of tools and strategies. J. Comput. Neurosci. 23, 349–398. 10.1007/s10827-007-0038-6 17629781 PMC2638500

[B12] Brunel N. (2000). Dynamics of sparsely connected networks of excitatory and inhibitory spiking neurons. J. Comput. Neurosci. 8, 183–208. 10.1023/a:1008925309027 10809012

[B13] Carnevale N. T. Hines M. L. (2006). The NEURON Book. Cambridge: Cambridge University Press.

[B14] Cartiglia M. Haessig G. Indiveri G. (2020). An error-propagation spiking neural network compatible with neuromorphic processors, in 2020 2nd IEEE International Conference on Artificial Intelligence Circuits and Systems (AICAS) (Genova: IEEE), 84–88.

[B15] Clopath C. Büsing L. Vasilaki E. Gerstner W. (2010). Connectivity reflects coding: a model of voltage-based STDP with homeostasis. Nat. Neurosci. 13, 344–352. 10.1038/nn.2479 20098420

[B16] Clopath C. Gerstner W. (2010). Voltage and spike timing interact in stdp–a unified model. Front. Synap. Neurosci. 2:25. 10.3389/fnsyn.2010.00025 21423511 PMC3059665

[B17] Davison A. Brüderle D. Eppler J. Kremkow J. Muller E. Pecevski D.. (2008). PyNN: a common interface for neuronal network simulators. Front. Neuroinformatics 2:11. 10.3389/neuro.11.011.2008 19194529 PMC2634533

[B18] D'Haene M. Hermans M. Schrauwen B. (2014). Toward unified hybrid simulation techniques for spiking neural networks. Neural Comput. 26, 1055–1079. 10.1162/NECO_a_00587 24684451

[B19] Diederich N. Bartsch T. Kohlstedt H. Ziegler M. (2018). A memristive plasticity model of voltage-based stdp suitable for recurrent bidirectional neural networks in the hippocampus. Sci. Rep. 8, 1–12. 10.1038/s41598-018-27616-6 29921840 PMC6008480

[B20] Djurfeldt M. Davison A. P. Eppler J. M. (2014). Efficient generation of connectivity in neuronal networks from simulator-independent descriptions. Front. Neuroinformatics 8:43. 10.3389/fninf.2014.00043 24795620 PMC4001034

[B21] Djurfeldt M. Hjorth J. Eppler J. M. Dudani N. Helias M. Potjans T. C.. (2010). Run-time interoperability between neuronal network simulators based on the MUSIC framework. Neuroinformatics 8, 43–60. 10.1007/s12021-010-9064-z 20195795 PMC2846392

[B22] Eppler J. M. Helias M. Muller E. Diesmann M. Gewaltig M. (2009). PyNEST: a convenient interface to the NEST simulator. Front. Neuroinformatics 2:12. 10.3389/neuro.11.012.2008 19198667 PMC2636900

[B23] Friedmann S. Schemmel J. Grübl A. Hartel A. Hock M. Meier K. (2016). Demonstrating hybrid learning in a flexible neuromorphic hardware system. IEEE Trans. Biomed. Circuits Syst. 11, 128–142. 10.1109/TBCAS.2016.2579164 28113678

[B24] Galluppi F. Lagorce X. Stromatias E. Pfeiffer M. Plana L. A. Furber S. B.. (2015). A framework for plasticity implementation on the spinnaker neural architecture. Front. Neurosci. 8:429. 10.3389/fnins.2014.00429 25653580 PMC4299433

[B25] Gerstner W. Kempter R. van Hemmen J. L. Wagner H. (1996). A neuronal learning rule for sub-millisecond temporal coding. Nature 383, 76–78. 10.1038/383076a0 8779718

[B26] Gerstner W. Kistler W. M. Naud R. Paninski L. (2014). Neuronal Dynamics. From single Neurons to Networks and Models of Cognition. Cambridge: Cambridge University Press.

[B27] Gewaltig M.-O. Diesmann M. (2007). NEST (NEural Simulation Tool). Scholarpedia 2:1430. 10.4249/scholarpedia.143019198667

[B28] Hahne J. Helias M. Kunkel S. Igarashi J. Bolten M. Frommer A.. (2015). A unified framework for spiking and gap-junction interactions in distributed neuronal network simulations. Front. Neuroinformatics 9:22. 10.3389/fninf.2015.00022 26441628 PMC4563270

[B29] Hannun A. Case C. Casper J. Catanzaro B. Diamos G. Elsen E.. (2014). Deep speech: Scaling up end-to-end speech recognition. arXiv [Preprint]. arXiv:1412.5567v2.

[B30] Hebb D. O. (1949). The Organization of Behavior: A Neuropsychological Theory. New York, NY: John Wiley & Sons.

[B31] Hines M. L. Carnevale N. T. (2001). NEURON: a tool for neuroscientists. Neuroscientist 7, 123–135. 10.1177/107385840100700207 11496923

[B32] Hines M. L. Morse T. Migliore M. Carnevale N. T. Shepherd G. M. (2004). ModelDB: A database to support computational neuroscience. J. Comput. Neurosci. 17, 7–11. 10.1023/B:JCNS.0000023869.22017.2e 15218350 PMC3732827

[B33] Hinton G. E. Osindero S. Teh Y.-W. (2006). A fast learning algorithm for deep belief nets. Neural Comput. 18, 1527–1554. 10.1162/neco.2006.18.7.1527 16764513

[B34] Jordan J. Helias M. Diesmann M. Kunkel S. (2020a). Efficient communication in distributed simulations of spiking neuronal networks with gap junctions. Front. Neuroinformatics 14:12. 10.3389/fninf.2020.00012 32431602 PMC7214808

[B35] Jordan J. Ippen T. Helias M. Kitayama I. Sato M. Igarashi J.. (2018). Extremely scalable spiking neuronal network simulation code: from laptops to exascale computers. Front. Neuroinformatics 12:2. 10.3389/fninf.2018.00002 PMC5820465 29503613

[B36] Jordan J. Mørk H. Vennemo S. B. Terhorst D. Peyser A. Ippen T.. (2019). NEST 2.18.0. 10.5281/zenodo.2605422

[B37] Jordan J. Schmidt M. Senn W. Petrovici M. A. (2020b). Evolving to learn: discovering interpretable plasticity rules for spiking networks. arXiv [Preprint]. arXiv:2005.14149v3.

[B38] Jülich Supercomputing Centre (2015). JUQUEEN: IBM Blue Gene/Q® Supercomputer System at the Jülich Supercomputing Centre.

[B39] Ko H. Cossell L. Baragli C. Antolik J. Clopath C. Hofer S. B.. (2013). The emergence of functional microcircuits in visual cortex. Nature 496, 96–100. 10.1038/nature12015 23552948 PMC4843961

[B40] Krishnan J. Porta Mana P. Helias M. Diesmann M. Di Napoli E. A. (2017). Perfect detection of spikes in the linear sub-threshold dynamics of point neurons. Front. Neuroinformatics 11:75. 10.3389/fninf.2017.00075 29379430 PMC5770835

[B41] Krizhevsky A. Sutskever I. Hinton G. E. (2012). Imagenet classification with deep convolutional neural networks, in Advances in Neural information Processing Systems (Tahoe, CA), 1097–1105.

[B42] Kumar N. M. Gilula N. B. (1996). The gap junction communication channel. Cell 84, 381–388. 10.1016/S0092-8674(00)81282-9 8608591

[B43] Kunkel S. (2015). Simulation technology for plastic neuronal networks on high- performance computers (Doctoral dissertation). FreiDok plus Universitätsbibliothek Freiburg, University of Freiburg, Freiburg, Germany. 10.6094/UNIFR/10419

[B44] Kunkel S. Diesmann M. Morrison A. (2011). Limits to the development of feed-forward structures in large recurrent neuronal networks. Front. Comput. Neurosci. 4:160. 10.3389/fncom.2010.00160 21415913 PMC3042733

[B45] Lecun Y. (1985). Une procedure d'apprentissage pour reseau a seuil asymmetrique (a learning scheme for asymmetric threshold networks), in Proceedings of Cognitiva 85 (Paris), 599–604.

[B46] LeCun Y. Bengio Y. Hinton G. (2015). Deep learning. Nature 521, 436–444. 10.1038/nature14539 26017442

[B47] Legenstein R. Maass W. (2011). Branch-specific plasticity enables self-organization of nonlinear computation in single neurons. J. Neurosci. 31, 10787–10802. 10.1523/JNEUROSCI.5684-10.2011 21795531 PMC6623094

[B48] Litwin-Kumar A. Doiron B. (2014). Formation and maintenance of neuronal assemblies through synaptic plasticity. Nat. Commun. 5, 1–12. 10.1038/ncomms6319 25395015

[B49] Lytton W. W. Seidenstein A. H. Dura-Bernal S. McDougal R. A. Schürmann F. Hines M. L. (2016). Simulation neurotechnologies for advancing brain research: parallelizing large networks in NEURON. Neural Comput. 28, 2063–2090. 10.1162/neco_a_00876 27557104 PMC5295685

[B50] Maes A. Barahona M. Clopath C. (2020). Learning spatiotemporal signals using a recurrent spiking network that discretizes time. PLoS Comput. Biol. 16:e1007606. 10.1371/journal.pcbi.1007606 31961853 PMC7028299

[B51] Markram H. Lübke J. Frotscher M. Sakmann B. (1997). Regulation of synaptic efficacy by coincidence of postsynaptic APs and EPSPs. Science 275, 213–215. 10.1126/science.275.5297.213 8985014

[B52] Markram H. Muller E. Ramaswamy S. Reimann M. W. Abdellah M. Sanchez C. A.. (2015). Reconstruction and simulation of neocortical microcircuitry. Cell 163, 456–492. 10.1016/j.cell.2015.09.029 26451489

[B53] Mayr C. G. Partzsch J. (2010). Rate and pulse based plasticity governed by local synaptic state variables. Front. Synapt. Neurosci. 2:33. 10.3389/fnsyn.2010.00033 21423519 PMC3059700

[B54] Moradi S. Qiao N. Stefanini F. Indiveri G. (2018). A scalable multicore architecture with heterogeneous memory structures for dynamic neuromorphic asynchronous processors (DYNAPs). IEEE Trans. Biomed. Circuits Syst. 12, 106–122. 10.1109/tbcas.2017.2759700 29377800

[B55] Morrison A. Aertsen A. Diesmann M. (2007a). Spike-timing dependent plasticity in balanced random networks. Neural Comput. 19, 1437–1467. 10.1162/neco.2007.19.6.1437 17444756

[B56] Morrison A. Diesmann M. (2008). Maintaining causality in discrete time neuronal network simulations, in Lectures in Supercomputational Neurosciences: Dynamics in Complex Brain Networks, eds Graben P. B. Zhou C. Thiel M. Kurths J. (Berlin; Heidelberg: Springer), 267–278.

[B57] Morrison A. Diesmann M. Gerstner W. (2008). Phenomenological models of synaptic plasticity based on spike-timing. Biol. Cybern. 98, 459–478. 10.1007/s00422-008-0233-1 18491160 PMC2799003

[B58] Morrison A. Mehring C. Geisel T. Aertsen A. Diesmann M. (2005). Advancing the boundaries of high connectivity network simulation with distributed computing. Neural Comput. 17, 1776–1801. 10.1162/0899766054026648 15969917

[B59] Morrison A. Straube S. Plesser H. E. Diesmann M. (2007b). Exact subthreshold integration with continuous spike times in discrete-time neural network simulations. Neural Comput. 19, 47–79. 10.1162/neco.2007.19.1.47 17134317

[B60] Muller E. Bednar J. A. Diesmann M. Gewaltig M.-O. Hines M. Davison A. P. (2015). Python in neuroscience. Front. Neuroinformatics 9:11. 10.3389/fninf.2015.00011 25926788 PMC4396193

[B61] Neftci E. Das S. Pedroni B. Kreutz-Delgado K. Cauwenberghs G. (2014). Event-driven contrastive divergence for spiking neuromorphic systems. Front. Neurosci. 7:272. 10.3389/fnins.2013.00272 24574952 PMC3922083

[B62] Neftci E. O. Augustine C. Paul S. Detorakis G. (2017). Event-driven random back-propagation: enabling neuromorphic deep learning machines. Front. Neurosci. 11:324. 10.3389/fnins.2017.00324 28680387 PMC5478701

[B63] Ngezahayo A. Schachner M. Artola A. (2000). Synaptic activity modulates the induction of bidirectional synaptic changes in adult mouse hippocampus. J. Neurosci. 20, 2451–2458. 10.1523/JNEUROSCI.20-07-02451.2000 10729325 PMC6772243

[B64] Parker D. (1985). Learning Logic. Technical Report TR-47.

[B65] Pecevski D. Kappel D. Jonke Z. (2014). Nevesim: event-driven neural simulation framework with a python interface. Front. Neuroinformatics 8:70. 10.3389/fninf.2014.00070 25177291 PMC4132371

[B66] Pfeil T. Grübl A. Jeltsch S. Müller E. Müller P. Petrovici M. A.. (2013). Six networks on a universal neuromorphic computing substrate. Front. Neurosci. 7:11. 10.3389/fnins.2013.00011 23423583 PMC3575075

[B67] Plesser H. Diesmann M. Gewaltig M.-O. Morrison A. (2015). NEST: the neural simulation tool, in Encyclopedia of Computational Neuroscience, eds Jaeger D. Jung R. (New York, NY: Springer), 1849–1852.

[B68] Plotnikov D. Blundell I. Ippen T. Eppler J. M. Morrison A. Rumpe B. (2016). NESTML: a modeling language for spiking neurons, in Modellierung 2016 Conference, Vol. 254 of LNI (Bonn: Bonner Köllen Verlag), 93–108

[B69] Qiao N. Mostafa H. Corradi F. Osswald M. Stefanini F. Sumislawska D.. (2015). A reconfigurable on-line learning spiking neuromorphic processor comprising 256 neurons and 128k synapses. Front. Neurosci. 9:141. 10.3389/fnins.2015.00141 25972778 PMC4413675

[B70] Ros E. Carrillo R. Ortigosa E. M. Barbour B. Agís R. (2006). Event-driven simulation scheme for spiking neural networks using lookup tables to characterize neuronal dynamics. Neural Comput. 18, 2959–2993. 10.1162/neco.2006.18.12.2959 17052155

[B71] Rudolph M. Destexhe A. (2006). Event-based simulation strategy for conductance-based synaptic interactions and plasticity. Neurocomputing 69, 1130–1133. 10.1016/j.neucom.2005.12.059

[B72] Rumelhart E. D. Geoffrey H. E. Ronald W. J. (1986). Learning representations by back-propagating errors. Nature 323, 533–536. 10.1038/323533a0

[B73] Sacramento J. Costa R. P. Bengio Y. Senn W. (2018). Dendritic cortical microcircuits approximate the backpropagation algorithm, in Advances in Neural Information Processing Systems (Montreal, QC), 8721–8732.

[B74] Sadeh S. Clopath C. Rotter S. (2015). Emergence of functional specificity in balanced networks with synaptic plasticity. PLoS Comput. Biol. 11:e1004307. 10.1371/journal.pcbi.1004307 26090844 PMC4474917

[B75] Schmidt M. Bakker R. Shen K. Bezgin G. Diesmann M. van Albada S. J. (2018). A multi-scale layer-resolved spiking network model of resting-state dynamics in macaque visual cortical areas. PLoS Comput. Biol. 14:e1006359. 10.1371/journal.pcbi.1006359 30335761 PMC6193609

[B76] Serrano-Gotarredona T. Masquelier T. Prodromakis T. Indiveri G. Linares-Barranco B. (2013). Stdp and stdp variations with memristors for spiking neuromorphic learning systems. Front. Neurosci. 7:2. 10.3389/fnins.2013.00002 23423540 PMC3575074

[B77] Sheik S. Paul S. Augustine C. Cauwenberghs G. (2016). Membrane-dependent neuromorphic learning rule for unsupervised spike pattern detection, in 2016 IEEE Biomedical Circuits and Systems Conference (BioCAS) (Shanghai: IEEE), 164–167.

[B78] Sjöström P. Turrigiano G. Nelson S. (2001). Rate, timing, and cooperativity jointly determine cortical synaptic plasticity. Neuron 32, 1149–1164. 10.1016/S0896-6273(01)00542-6 11754844

[B79] Song S. Miller K. D. Abbott L. F. (2000). Competitive Hebbian learning through spike-timing-dependent synaptic plasticity. Nat. Neurosci. 3, 919–926. 10.1038/78829 10966623

[B80] Song S. Sjöström P. Reigl M. Nelson S. Chklovskii D. (2005). Highly nonrandom features of synaptic connectivity in local cortical circuits. PLoS Biol. 3:e68. 10.1371/journal.pbio.0030068 15737062 PMC1054880

[B81] Stapmanns J. Hahne J. Helias M. Bolten M. Diesmann M. Dahmen D. (2021). Event-based update of synapses in voltage-based learning rules. Zenodo. 10.5281/zenodo.4565188 PMC8222618 34177505

[B82] Stimberg M. Goodman D. Benichoux V. Brette R. (2014). Equation-oriented specification of neural models for simulations. Front. Neuroinformatics 8:6. 10.3389/fninf.2014.00006 24550820 PMC3912318

[B83] Thakur C. S. Molin J. L. Cauwenberghs G. Indiveri G. Kumar K. Qiao N.. (2018). Large-scale neuromorphic spiking array processors: a quest to mimic the brain. Front. Neurosci. 12:891. 10.3389/fnins.2018.00891 30559644 PMC6287454

[B84] Urbanczik R. Senn W. (2014). Learning by the dendritic prediction of somatic spiking. Neuron 81, 521–528. 10.1016/j.neuron.2013.11.030 24507189

[B85] Watts L. (1994). Event-driven simulation of networks of spiking neurons, in Advances in Neural Information Processing Systems (Denver, CO), 927.

[B86] Werbos P. (1974). Beyond Regression: New Tools for Prediction and Analysis in the Behavioral Sciences. Cambridge, MA: Harvard University.

[B87] Yger P. Harris K. D. (2013). The convallis rule for unsupervised learning in cortical networks. PLoS Comput. Biol. 9:e1003272. 10.1371/journal.pcbi.1003272 24204224 PMC3808450

[B88] Zenke F. Gerstner W. (2014). Limits to high-speed simulations of spiking neural networks using general-purpose computers. Front. Neuroinformatics 8:76. 10.3389/fninf.2014.00076 25309418 PMC4160969

