# Supplementary material for: Event-Based Update of Synapses in Voltage-Based Learning Rules
Source: Front Neuroinform. 2021 Jun 10;15:609147. doi: 10.3389/fninf.2021.609147 (PMC8222618; doi:10.3389/fninf.2021.609147)
Supplement: Supplementary file 1 [file Table_1.pdf]

Event-based update of synapses in voltage-based learning rules: Supplementary Material

**A: Simulation parameters**		
**Symbol**	**Value**	**Description**
f_{pair}	$[10,\ 11, \ldots, 50]$ Hz	frequency of occurrence of spike pairs
Δt	$\pm 10\,\text{ms}$	time shift of spike pair
w_{init} [mV]	0.5 mV or pA/ms	initial weight (unit is mV for aeif and pA/ms for hh neuron)
**B: Parameters of `aeif_psc_delta_clopath`**		
**Symbol**	**Value**	**Description**
E_L	$-54.402\,\text{mV}$	leak reversal potential
E_{Na}	$50.0\,\text{mV}$	sodium reversal potential
E_{K}	$-77.0\,\text{mV}$	potassium reversal potential
g_L	$30.0\,\text{nS}$	leak conductance
g_{Na}	$12 \cdot 10^3\,\text{nS}$	sodium peak conductance
g_{K}	$3.6 \cdot 10^3\,\text{nS}$	potassium peak conductance
C_m	$100\,\text{pF}$	membrane capacitance
τ_{ex}	$0.2\,\text{ms}$	rise time of the exc. synaptic alpha funct.
τ_{in}	$2.0\,\text{ms}$	rise time of the inh. synaptic alpha funct.
θ_-	$-64.9\,\text{mV}$	threshold
θ_+	$-35\,\text{mV}$	threshold
A_{LTD}	$1 \cdot 10^{-4}\,1/\text{mV}$	amplitude of LTD
A_{LTP}	$12 \cdot 10^{-4}\,1/\text{mV}^2$	amplitude of LTP
τ_-	$10\,\text{ms}$	time constant of $\bar{u}_-$
τ_+	$114\,\text{ms}$	time constant of $\bar{u}_+$
τ_s	$15\,\text{ms}$	time constant of s_j^*
d_s	$5\,\text{ms}$	delay of $\bar{u}_\pm$
**C: Parameters of `hh_psc_alpha_clopath`**		
**Symbol**	**Value**	**Description**
E_L	$-70.6\,\text{mV}$	leak reversal potential
g_L	$30\,\text{nS}$	leak conductance
C_m	$281\,\text{pF}$	membrane capacitance
V_{reset}	$-60\,\text{mV}$	reset value of membr. pot. after spike
V_{peak}	$33\,\text{mV}$	spike detection threshold
Δ_T	$2\,\text{mV}$	slope factor
τ_w	$144\,\text{ms}$	spike adaptation time constant
τ_z	$40\,\text{ms}$	spike adaptation time constant
$V_{\text{th,max}}$	$30.4\,\text{mV}$	threshold potential after spike
$\tau_{V,\text{th}}$	$50\,\text{ms}$	threshold potential time constant
a	$4\,\text{nS}$	subthreshold adaptation
b	$0.0805\,\text{pA}$	spike triggered adaptation
θ_-	$-70.6\,\text{mV}$	threshold of $\bar{u}_-$
θ_+	$-45.3\,\text{mV}$	threshold of $\bar{u}_+$
A_{LTD}	$14 \cdot 10^{-5}\,1/\text{mV}$	amplitude of LTD
A_{LTP}	$8 \cdot 10^{-5}\,1/\text{mV}^2$	amplitude of LTP
τ_-	$10\,\text{ms}$	time constant of $\bar{u}_-$
τ_+	$7\,\text{ms}$	time constant of $\bar{u}_+$
τ_s	$15\,\text{ms}$	time constant of s_j^*
d_s	$4\,\text{ms}$	delay of $\bar{u}_\pm$

Table S1. Parameters of the spike pairing experiment using the Clopath rule. The values for the aeif model are taken from (**Clopath et al.**, 2010, Tab. 1 and appendix) and those for the hh model are extracted from the reference implementation by B. Torben-Nielson on ModelDB (**Hines et al.**, 2004).

**A: Model summary**	
**Populations**	Three: excitatory, inhibitory, external input
**Connectivity**	all-to-all, fixed out-degree, fixed in-degree
**Neuron model**	adaptive exponential integrate-and-fire (aeif, Clopath)
**Plasticity**	Clopath synapse/
**Input**	independent homogeneous Poisson spike trains
**Measurements**	synapse weight

B: Populations

**Name**	**Elements**	**Population size**
E	aeif/two-comp.	$N_E = 10$
I	aeif/two-comp.	$N_I = 3$
E_{ext}	Poisson generator	$N_p = 500$

C: Connectivity

**Name**	**Source**	**Target**	**Pattern**
ExcExc	E	E	all-to-all (no autapses)
ExcInh	E	I	fixed in-degree $C_E = 8$
InhExc	I	E	fixed out-degree $C_I = 6$
ExtExc	E_{Ext}	E	all-to-all
ExtInh	E_{Ext}	I	all-to-all

D: Neurons

**Name**	`aeif_psc_delta_clopath`
**Type**	adaptive exponential integrate-and-fire
**Details**	see **Clopath et al.** (2010)
**Parameters**	see Table S3

E: Synapses

**Name**	**Model**	**Initial weight [mV]**	**Max. weight [mV]**
ExcExc	`clopath_synapse`	0.25	0.75
ExcInh	`static_synapse`	1.0	–
InhExc	`static_synapse`	1.0	–
ExtExc	`clopath_synapse`	random uniform from $[0.5, 1.5]$	3.0
ExtInh	`static_synapse`	random uniform from $[0.0, 0.5]$	–

F: Input

**Type**	Poisson generator
**Description**	homogeneous Poisson spike trains, independent for each neuron, modulated rate
**Parameters**	see Table S3

Table S2. Model description of a small excitatory-inhibitory network after Nordlie et al. (2009). This network reproduces the emergence of strong bidirectional couplings using the Clopath rule shown in Figure 7 . The values of the parameters are shown in Table S3.

A: Parameters of `aeif_psc_delta_clopath`		
**Symbol**	**Value**	**Description**
E_L	$-70.6\,\mathrm{mV}$	leak reversal potential
g_L	$30\,\mathrm{nS}$	leak conductance
C_m	$281\,\mathrm{pF}$	membrane capacitance
V_{reset}	$-60\,\mathrm{mV}$	reset value of membr. pot. after spike
V_{peak}	$20\,\mathrm{mV}$	spike detection threshold
Δ_T	$2\,\mathrm{mV}$	slope factor
τ_w	$144\,\mathrm{ms}$	spike adaptation time constant
τ_z	$40\,\mathrm{ms}$	spike adaptation time constant
$V_{\mathrm{th,max}}$	$30.4\,\mathrm{mV}$	threshold potential after spike
$\tau_{V,\mathrm{th}}$	$50\,\mathrm{ms}$	threshold potential time constant
a	$4\,\mathrm{nS}$	subthreshold adaptation
b	$0.0805\,\mathrm{pA}$	spike triggered adaptation
θ_-	$-70.6\,\mathrm{mV}$	threshold of $\bar{u}_-$
θ_+	$-45.3\,\mathrm{mV}$	threshold of $\bar{u}_+$
A_{LTD}	$14 \cdot 10^{-5}\,1/\mathrm{mV}$	amplitude of LTD
u_{ref}	60	reference value for $\bar{\bar{u}}$
$\bar{\bar{\tau}}$	$1.5 \cdot 10^{3}\,\mathrm{ms}$	time constant of $\bar{\bar{u}}$
A_{LTP}	$8 \cdot 10^{-5}\,1/\mathrm{mV}^2$	amplitude of LTP
τ_-	$10\,\mathrm{ms}$	time constant of $\bar{u}_-$
τ_+	$7\,\mathrm{ms}$	time constant of $\bar{u}_+$
τ_s	$15\,\mathrm{ms}$	time constant of s_j^*
d_s	$4\,\mathrm{ms}$	delay of $\bar{u}_\pm$
**B: Input parameters**		
**Symbol**	**Value**	**Description**
A_p	66 [Hz]	amplitude of Gaussian rate profile
σ_p	10	width of Gaussian rate profile
c_p	0.48 [Hz]	offset of Gaussian rate profile
s_p	$[25, 75, \ldots, 475]$	set of possible values for the center of the Gaussian μ_p
t_μ	100 [ms]	interval after which a new value for μ_p is drawn
N_μ	100	number of intervals t_μ

Table S3. Neuron and input parameters for simulation of network producing bidirectional connections using the Clopath rule. The values are taken from (**Clopath et al.**, 2010, Tab. 1 and appendix). The same values are used for the performance measurements shown in Figures 9 and 10.

**A: Simulation parameters**		
**Symbol**	**Value**	**Description**
T_p	$1000\,\mathrm{ms}$	pattern duration
N_{rep}	100	number of pattern repetitions
N_{p}	200	number of input spike trains
f_{p}	$10\,\mathrm{Hz}$	input firing rate
w_{g_E}	$(18\,\sin(2\pi t) + 4.8)\ \mathrm{nS}$	weights to generate periodic excitatory conductance
w_{g_I}	$3\,\mathrm{nS}$	weights to generate constant inhibitory conductance
**B: Parameters of `pp_cond_exp_mc_urbanczik (soma)`**		
**Symbol**	**Value**	**Description**
C_m	$300\,\mathrm{pF}$	membrane capacitance
E_L	$-70\,\mathrm{mV}$	leak reversal potential
g_L	$30.0\,\mathrm{nS}$	leak conductance
E_{ex}	$0.0\,\mathrm{mV}$	exc. reversal potential
E_{in}	$-75.0\,\mathrm{mV}$	inh. reversal potential
τ_{ex}	$3.0\,\mathrm{ms}$	rise time of the exc. synaptic alpha funct.
τ_{in}	$3.0\,\mathrm{ms}$	rise time of the inh. synaptic alpha funct.
t_{ref}	$3.0\,\mathrm{ms}$	refractory time
**C: Parameters of `pp_cond_exp_mc_urbanczik (dendrite)`**		
**Symbol**	**Value**	**Description**
C_m	$300\,\mathrm{pF}$	membrane capacitance
E_L	$-70\,\mathrm{mV}$	leak reversal potential
g_L	$30.0\,\mathrm{nS}$	leak conductance
τ_{ex}	$3.0\,\mathrm{ms}$	rise time of the exc. synaptic alpha funct.
τ_{in}	$3.0\,\mathrm{ms}$	rise time of the inh. synaptic alpha funct.
$\phi(U)$	$\frac{0.15\,\mathrm{kHz}}{1+\frac{1}{2}\exp\left(\frac{-55\,\mathrm{mV}-U}{3\,\mathrm{mV}}\right)}$	rate function
g_{sp}	$600.0\,\mathrm{nS}$	coupling dendrite to soma

Table S4. Parameters of the simulation of the learning experiment using the Urbanczik-Senn rule. The same values of the neuron parameters are used for the performance measurements shown in Figures 11 and 12.

A: Model summary

**Populations**	Three: excitatory, inhibitory, external input
**Connectivity**	random with fixed indegree
**Neuron model**	adaptive exponential integrate-and-fire (aeif, Clopath)/ two-compartment Poisson (two-comp., Urbanczik-Senn)
**Plasticity**	Clopath synapse/ Urbanczik-Senn synapse
**Input**	independent homogeneous Poisson spike trains
**Measurements**	—

B: Populations

**Name**	**Elements**	**Population size**
E	aeif/two-comp.	$N_E = 4N_I$
I	aeif/two-comp.	N_I
E_{ext}	Poisson generator	1

C: Connectivity

**Name**	**Source**	**Target**	**Pattern**	**Weight**
Exc	E	E+I	fixed in-degree C_E	J_{ex}
Inh	I	E+I	fixed in-degree C_I	J_{in}
Ext	E_{Ext}	E+I	all-to-all	J

D: Neurons

**Name**	`aeif_psc_delta_clopath`
**Type**	adaptive exponential integrate-and-fire
**Details**	see **Clopath et al.** (2010)
**Parameters**	see Table S3
**Name**	`pp_cond_exp_mc_urbanczik`
**Type**	two-compartment neuron with spike generation via inhomogeneous Poisson process
**Details**	see **Urbanczik and Senn** (2014)
**Parameters**	see Table S4

E: Synapses

**Name**	**Model**
Exc	`clopath/urbanczik_synapse`
Inh	`clopath/urbanczik_synapse`
Ext	`static_synapse`

F: Input

**Type**	**Description**
Poisson generator	homogeneous Poisson spike trains, independent for each neuron, rate $f_{ext} = \nu_{ext} C_E$

Table S5. Model description of Brunel network after Nordlie et al. (2009). This network is used to produce the performance measurement shown in Figures 9, 10, 11, and 12. The values of the parameters are shown in Table S6.

A: Global simulation parameters		
**Symbol**	**Value**	**Description**
T	$2 \cdot 10^3\,\mathrm{ms}$	biological time
h	$0.1\,\mathrm{ms}$	resolution
**B: Network sizes**		
**Symbol**	**Value**	**Description**
$N = N_E + N_I$	$1.92 \cdot 10^6$	number of neurons in Clopath simulation with small indegree $K = 100$
N	$1.54 \cdot 10^5$	number of neurons in Clopath simulation with large indegree $K = 5000$
N	$3.84 \cdot 10^5$	number of neurons in Urbanczik simulation with small indegree $K = 100$
N	$3.84 \cdot 10^4$	number of neurons in Urbanczik simulation with large indegree $K = 5000$
**C: Connectivity**		
**Symbol**	**Value**	**Description**
g	7.0	ratio inh./exc. weight
J	0.1	postsynaptic amplitude The unit depends on the neuron model. In case of the aeif model and the Clopath rule it is $[\mathrm{mV}]$ and for the Urbanczik-Senn rule it is $[\mathrm{pA}]$
J_{ex}	J	amplitude of exc. postsyn. potential
J_{in}	$-gJ_{\mathrm{ex}}$	amplitude of inh. postsyn. potential
$K = C_E + C_I$	100 or 5000	total number of excitatory synapses per neuron
C_E	$0.8K$	number of excitatory synapses per neuron
C_I	$0.2K$	number of inhibitory synapses per neuron
η	0.0	learning rate
**D: External input**		
**Symbol**	**Value**	**Description**
ν_{ext}	$6.75 \cdot 10^{-3}\,\mathrm{Hz}$	factor in rate of external Poisson input $f_{\mathrm{ext}} = \nu_{\mathrm{ext}} C_E$

Table S6. Parameters of the Brunel network.

REFERENCES

Clopath, C., Büsing, L., Vasilaki, E., and Gerstner, W. (2010), Connectivity reflects coding: a model of voltage-based STDP with homeostasis, *Nat. Neurosci.*, 13, 344–352

Hines, M. L., Morse, T., Migliore, M., Carnevale, N. T., and Shepherd, G. M. (2004), ModelDB: A database to support computational neuroscience, *J. Comput. Neurosci.*, 17, 1, 7–11, doi:10.1023/B:JCNS.0000023869.22017.2e

Nordlie, E., Gewaltig, M.-O., and Plesser, H. E. (2009), Towards reproducible descriptions of neuronal network models, *PLOS Comput. Biol.*, 5, 8, e1000456, doi:10.1371/journal.pcbi.1000456

Urbanczik, R. and Senn, W. (2014), Learning by the dendritic prediction of somatic spiking, *Neuron*, 81, 3, 521–528
